# The relationship between atmospheric particulate matter, leaf surface microstructure, and the phyllosphere microbial diversity of *Ulmus* L.

**DOI:** 10.1186/s12870-024-05232-z

**Published:** 2024-06-17

**Authors:** Liren Xu, Yichao Liu, Shuxiang Feng, Chong Liu, Xinyu Zhong, Yachao Ren, Yujun Liu, Yinran Huang, Minsheng Yang

**Affiliations:** 1 Hebei Agricultural University, Baoding, 071000 Hebei China; 2 Hebei Academy of Forestry and Grassland Science, Shijiazhuang, 050061 Hebei China; 3 National Engineering Laboratory for Tree Breeding, College of Biological Sciences and Biotechnology, Beijing Forestry University, Beijing, 100083 China

**Keywords:** Elm, Atmospheric pollution, Phyllosphere microbiome, PM-borne microorganisms, Foliar microstructures

## Abstract

**Background:**

Plants can retain atmospheric particulate matter (PM) through their unique foliar microstructures, which has a profound impact on the phyllosphere microbial communities. Yet, the underlying mechanisms linking atmospheric particulate matter (PM) retention by foliar microstructures to variations in the phyllosphere microbial communities remain a mystery. In this study, we conducted a field experiment with ten *Ulmus* lines. A series of analytical techniques, including scanning electron microscopy, atomic force microscopy, and high-throughput amplicon sequencing, were applied to examine the relationship between foliar surface microstructures, PM retention, and phyllosphere microbial diversity of *Ulmus* L.

**Results:**

We characterized the leaf microstructures across the ten *Ulmus* lines. Chun exhibited a highly undulated abaxial surface and dense stomatal distribution. Langya and Xingshan possessed dense abaxial trichomes, while Lieye, Zuiweng, and Daguo had sparsely distributed, short abaxial trichomes. Duomai, Qingyun, and Lang were characterized by sparse stomata and flat abaxial surfaces, whereas Jinye had sparsely distributed but extensive stomata. The mean leaf retention values for total suspended particulate (TSP), PM_2.5_, PM_2.5-10_, PM_10-100_, and PM_> 100_ were 135.76, 6.60, 20.10, 90.98, and 13.08 µg·cm^− 2^, respectively. Trichomes substantially contributed to PM_2.5_ retention, while larger undulations enhanced PM_2.5-10_ retention, as evidenced by positive correlations between PM_2.5_ and abaxial trichome density and between PM_2.5-10_ and the adaxial raw microroughness values. Phyllosphere microbial diversity patterns varied among lines, with bacteria dominated by *Sediminibacterium* and fungi by *Mycosphaerella*, *Alternaria*, and *Cladosporium*. Redundancy analysis confirmed that dense leaf trichomes facilitated the capture of PM_2.5_-associated fungi, while bacteria were less impacted by PM and struggled to adhere to leaf microstructures. Long and dense trichomes provided ideal microhabitats for retaining PM-borne microbes, as evidenced by positive feedback loops between PM_2.5_, trichome characteristics, and the relative abundances of microorganisms like *Trichoderma* and *Aspergillus*.

**Conclusions:**

Based on our findings, a three-factor network profile was constructed, which provides a foundation for further exploration into how different plants retain PM through foliar microstructures, thereby impacting phyllosphere microbial communities.

**Supplementary Information:**

The online version contains supplementary material available at 10.1186/s12870-024-05232-z.

## Background

Rapid industrial development and urbanization have led to the emission of large amounts of harmful gases and particulate matter (PM) into the atmosphere [1], which has been particularly pronounced in developing countries [2]. Atmospheric PM, especially PM_2.5_ (diameter ≤ 2.5 μm) and PM_10_ (diameter ≤ 10 μm), often contains heavy metals and can lead to urban haze, which has become the most serious air pollution issue in China in recent decades [3, 4]. Numerous studies have shown that plants can improve air quality in polluted areas by filtering PM from the air and retaining it on their unique leaf surface microstructures [5, 6]. This process involves a complex mechanism, with a plant’s ability to retain PM affected not only by its physicochemical properties but also by simultaneous interactions among different PM sources [7, 8] and meteorological conditions (e.g., wind and precipitation) [9].

Leaves are the most dominant component of the phyllosphere [10], one of the most diverse ecosystems on Earth [11], and an ideal environment for investigating plant growth and development [12]. Phyllosphere microorganisms, defined as microbes that reside on the surface of plant leaves [13], including bacteria, fungi, and other microorganisms, form complex communities on leaf surfaces [10]. These microorganisms are commonly deposited by natural factors [14], such as wind, raindrops [15], insects [16], or aerosols [17], owing to the unique microstructures of the leaf surface. PM also exerts impacts on the phyllosphere microbial communities [12]. For example, researchers have found that the phyllosphere microbiome of *Platanus sp*. L. trees is partially driven by PM [18]. Another recent study showed that progressively rising PM pollution levels enhance the phyllosphere microbial diversity of *Quercus ilex* L [19]. However, whether the microorganisms carried by PM, either bacteria or fungi or both, are specifically enriched by a particular leaf surface microstructure remains unclear and needs to be determined using high-throughput amplicon sequencing and advanced electron microscopy techniques.

The genus *Ulmus* L. (family Ulmaceae Mirb.) comprises about 40 species, of which 25 species, six varieties, and three cultivars are recorded in China. As a perennial deciduous woody plant, *Ulmus* species are widely distributed in the temperate regions of the Northern Hemisphere; in China, they are mainly distributed and cultivated in the vast temperate region north of the Yangtze River [20, 21]. Many *Ulmus* trees and shrubs are popular afforestation, timber, and landscaping species for their adaptability to different environmental conditions [22–24].

In this study, to elucidate the underlying relationship between the PM, foliar microstructures, and phyllosphere microbial diversity, we used ten asexual lines (to ensure a consistent genetic background) of eight distinct species, one variety, and one cultivar of *Ulmus* as study materials. Sanning electron microscopy (SEM) and atomic force microscopy (AFM) were applied to characterize the foliar microstructures. Using the laser particle size, we further characterized the particle size distribution of the PM retained on the foliar surface. Furthermore, we used 16 S rRNA gene and ITS high-throughput amplicon sequencing to analyze the phyllosphere microbial diversity among the lines (consist of bacteria and fungi). Based on the unique foliar microstructures of plants and their known role in retaining PM, we hypothesize that specific variations in leaf microstructures would influence the phyllosphere microbial community by providing distinct microhabitats for PM-borne microorganisms. To address this hypothesis, our study was designed to investigate: (1) how foliar surface microstructures influence PM retention capacity, and (2) how variations in PM retention shape the diversity and composition of phyllosphere microorganisms across the ten *Ulmus* lines.

## Materials and methods

### Study site and plant materials

The study site (38.1517°N, 114.4843°E) was a nursery at Hebei Academy of Forestry and Grassland Science located in Xinhua district, Shijiazhuang, Hebei Province, China, that was surrounded by schools, parks, and residential areas [25] (Fig. S1). The study site has a typical temperate continental monsoon climate, an average annual temperature of 12.9 °C, and an average annual precipitation of 550 mm. According to monitoring data obtained from the Qingyue Database, the local average daily PM_2.5_ concentration from January 2020 to December 2022 was 50.63 µg·m^− 3^ (https://data.epmap.org), which exceeded the secondary concentration limit defined by the Chinese National Ambient Air Quality Standard (GB3095-2012) [26].

The experimental saplings were planted in the study site in 2020 and were grafted from ten *Ulmus* asexual lines (for leaf characteristic details of the ten lines, please see Table S1): *U. davidiana* Planch. var. *japonica* (Rehd.) Nakai (referred to hereinafter as Chun), *U. pumila* L. cv. ‘Jinye’ (Jinye), *U*. *gaussenii* Cheng (Zuiweng), *U*. *chenmoui* Cheng (Langya), *U*. *macrocarpa* Hance (Daguo), *U*. *pumila* L. (Qingyun), *U*. *parvifolia* Jacq. (Lang), *U*. *castaneifolia* Hemsl. (Duomai), *U*. *laciniata* (Trautv.) Mayr. (Lieye), and *U*. *bergmanniana* Schneid. (Xingshan). Each line was planted in a row of eight plants in a north–south direction, with a spacing between plants and rows of 2.5 m. Identical water and fertilizer management strategies were applied.

### Sampling of leaves

Three individual plants (i.e., three biological replicates) with consistent growth and free of pests and diseases were selected in July 2022 from each of the ten *Ulmus* lines (height of 3.14 ± 0.12 m and ground diameter of 9.51 ± 1.21 cm). Approximately 300 leaf blades of each plant line were randomly collected from different directions with stainless steel scissors and mixed into germ-free sampling bags from 9:00 a.m.–11:00 a.m. on July 22, 2022. The leaf samples were stored in a 4℃ refrigerator for subsequent testing. Medical surgical masks, facial screens, and disposable nitrile gloves were worn during the sampling procedures. For each plant sample collected, the entire sampling instrument was disinfected with 75% medical ethanol.

### SEM and AFM assays

Ten leaf blades (i.e., 10 technical replicates) from each *Ulmus* line (each line consists of three biological replicates) mentioned in Section “Sampling of leaves” were chosen at random after sampling for 1 d. After 3 days of moisture removal with silica gel, three flat discs were taken from their marginal area with a 0.5-cm punch. These discs were gold-sprayed by an ion sputter coater (Ultim Max 65, Oxford Co., Ltd., UK) for 45 s, and then analyzed on both sides at various magnifications using SEM (SU8100, Hitachi, Co., Ltd., Tokyo, Japan) in low-vacuum mode (5.0 kV). The SEM images were imported into Image J software (ver. 1.51, National Institutes of Health, Bethesda, MD, USA) to calculate the trichome density, trichome length, stomata size, stomata area, and stomata density of the leaf surfaces of the ten *Ulmus* lines. Then, AFM (SPI3800N, Seiko Instruments, Co., Ltd., Tokyo, Japan) was applied to scan the surfaces of the discs with an Si_3_N_4_ nanoprobe. The maximum scan range, lateral resolution, vertical resolution, and scan rate were set to 0.5 Hz, 0.2 nm, 0.01 nm, and 5.0 × 5.0 μm, respectively. The AFM images were imported into NanoScope Analysis software (ver. 1.70, Bruker Corporation, Billerica, MA, USA) to accurately quantify the profile arithmetic average error (Ra), raw microroughness (RMS), and peak and valley (PV) values of the leaf surfaces for the ten *Ulmus* lines.

### Analysis of the total suspended particulate (TSP) retained on leaf blades

The measurement of TSP was conducted as reported by Tan et al. [6], with a slight modification. Briefly, ten leaf blades (10 technical replicates) from each of the ten *Ulmus* lines (each line consists of three biological replicates) were randomly selected, and cleaned in an ultrasonic device with 95% ethanol for 5 min. Then, a quantitative filter paper was used to gently absorb alcohol from the leaf surface for each leaf blade. Next, the leaf blades were left on an ultra-clean bench for 3 min to fully evaporate the alcohol from the leaf surface. Before and after cleaning, the weights of the leaf blades were measured using a 0.1‰-balance (BT125D, Sartorius, Göttingen, Germany) and recorded as *m*_1_ and *m*_2_, respectively. The blades were subsequently evenly placed on an A4-sized white paper sheet, then captured using a touch-free scanner (GP1600AF, Comet Co., Ltd., Guangdong, China), and the images were imported into Image J software to calculate the double-sided leaf area (S). These steps were repeated six times, and then the leaf retention TSP was calculated as follows: TSP = (*m*_1_ − *m*_2_) / S. The coefficient of variation (CV) of the TSP retention values (TSP–CV) served as a measure of the relative stability of foliar PM retention capacity among the ten *Ulmus* lines, and were calculated as follows: TSP–CV = TSP mean value/ standard deviation (SD).

### Particle size distribution assay

Fifteen blades from each of the ten *Ulmus* lines mentioned in Section “Sampling of leaves” were chosen at random after sampling for 1 d (15 technical replicates). The leaf blades were then rinsed separately with double-distilled water (ddH_2_O) several times before being collected in a 50-mL sterile centrifuge tube. Following ultrasonic oscillation for 30 s, the suspension was measured using a laser particle sizer (Mastersizer 2000, Malvern Panalytical, Co., Ltd., Malvern, UK), in accordance with the instrumental recommendations. The amount of suspension added to the instrument was determined by the real-time obscuration degree displayed by the Mastersizer 2000 software. The optimal laser obscuration was limited to 10–20%.

### Microbiome analysis of the leaf surface

Considering the complexity of biological samples, using a single leaf as a biological replicate may not adequately represent the phyllosphere microbial community. Therefore, we employed a composite sampling strategy to create a representative sample for each *Ulmus* line, which was then sequenced in triplicate. This approach helps capture the natural variability within the microbial community and provides a more accurate reflection of the community structure. For the leaf microbiome analysis, in detail, a total of 120 leaves from each of the ten *Ulmus* lines (each line consists of three individual plants) mentioned in Section “Sampling of leaves” were randomly selected after sampling for 1 d. Then 120 blades from each of the ten lines were divided equally into three parts (three technical replicates; each of the three parts consists of 40 leaves). Next, the leaf surface was rinsed with nuclease-free ddH_2_O to collect the suspension. DNA from the phyllosphere microorganisms was extracted using a soil DNA isolation kit (116560-200, MP Biomedicals, Santa Ana, CA, USA) according to the manufacturer’s instructions. The primers for bacterial (16 S rRNA gene, V3 + V4) and fungal (ITS, ITS1) rDNA amplification were as follows: 16 S rRNA gene: 5′-CCTACGGGNGGCWGCAG-3′ (341 F) and 5′-GGACTACHVGGGTAT-CTAAT-3′ (806R); ITS: 5′-TAGAGGAAGTAAAAGTCGTA-3′ (ITS1_F_KYO2) and 5′-TTCAAAGATTCG-ATGATTCAC-3′ (ITS86R) [27, 28].

The target sequences from the 16 S rRNA gene and ITS were amplified via polymerase chain reaction (PCR), with an initial denaturation step at 95 °C for 5 min, followed by 30 cycles (95 °C for 1 min, 60 °C for 1 min, and 72 °C for 1 min), with a final extension at 72 °C for 7 min. Subsequently, the PCR products were purified using AMPure XP Beads (Beckman, CA, USA) following the manufacturer’s instructions. Sequencing libraries were prepared using the Illumina DNA Prep Kit (Illumina, CA, USA), according to the manufacturer’s recommendations, and assessed using the ABI StepOnePlus Real-Time PCR System (Life Technologies, Foster City, USA). Finally, the qualified libraries were sequenced using the Illumina MiSeq platform (PE250, Illumina, San Diego, CA, USA) at Gene Denovo Biotechnology Co., Guangzhou, China.

The quality control of raw sequencing reads was performed using FastP software (ver. 0.18.0) [29] to remove (1) the reads containing more than 10% of unknown nucleotides (N); (2) the reads containing more than 50% of bases with quality (Q-value) < 20; and (3) the adapter contamination. This process ensures that only high-quality reads are retained for subsequent analysis, minimizing the impact of sequencing errors and artifacts. Following quality control, the clean reads were processed to merge paired-end reads into contiguous tags, using the FLASH software (ver. 1.2.11) [30]. Next, the method for filtering the raw tags was performed as reported by Bokulich et al. [31]. The operational taxonomic unit (OTU) method was more tolerant to sequencing errors and PCR artifacts compared to the amplicon sequence variants method [32]. Subsequently, clean tags with a sequence similarity of ≥ 97% were clustered into OTU using the UPARSE function within the USEARCH software (ver. 9.2.64) [33], and then matched to the NCBI 16 S rRNA gene database (ver. 202,101; http://www.ncbi.nlm.nih.gov) and UNITE ITS database (ver. 8.0) [34].

For alpha diversity analysis, Shannon and Pielou evenness indices were calculated in QIIME software (ver. 1.9.1) [35]. Alpha index comparison among groups was computed by Tukey’s test using the *vegan* package (ver. 2.5.3) [36] in R software (ver. 4.4.0). Principle component analysis (PCA) was also performed by using the *vegan* package (ver. 2.5.3) in R software (ver. 4.4.0). ADONIS function in the *vegan* package (ver. 2.5.3) was used to perform a permutational analysis of variance (PERMANOVA) to test the differences in the bacterial and fungal community composition among the ten *Ulmus* lines based on the Bray-Curtis distance measures (permutations = 999). Next, after filtering out the outlier and insignificant indicators (*p* > 0.05), the same R package (i.e., *vegan*; ver. 2.5.3) was used to conduct a redundancy analysis (RDA) for evaluating the correlation between environmental factors (i.e., foliar microstructures and PM) and phyllosphere microbial communities. PICRUSt (ver. 2.1.4) [37] and FunGuild (ver. 1.1) [38] software programs were used to predict the function of the bacterial and fungal communities, respectively. The 24 representative microorganisms (12 each of bacteria and fungi) for the microbial community were selected based on their known ecological roles and functional importance, as documented in the cited references [39–61] (for details, please see Table S2).

### Statistical analysis

All experiments in this study were conducted with at least three biological replicates. The number of measurements for each index in each experimental replicate was given in the previous subsections. The SPSS software (ver. 25.0, IMB Corp., Armonk, NY, USA) was used for all statistical analyses. Following the confirmation of normality for all datasets via the Kolmogorov-Smirnov test, a one-way analysis of variance (ANOVA) was performed to evaluate whether there was significance difference among the samples. The assumption of homogeneity of variances was confirmed using Levene’s test, indicating homogeneous variances among groups (*p* > 0.05). Then Tukey’s test was employed to conduct post hoc pairwise comparisons among all samples (*p* < 0.05). Multiple regression analysis was applied to examine the potential relationship between phyllosphere microorganisms, PM, and leaf microstructures using the SPSS software (ver. 25.0). GraphPad Prism (ver. 8.0, GraphPad Software Corporation, San Diego, CA, USA) and RStudio (ver. 2021.09.0 Build 35, Posit Software Corporation, Boston, MA, USA) were used to visualize the data. A flowchart was presented in Fig. S2 to better visualize the experimental processes for this study.

## Results

### The morphology and microstructure of the leaf surface varied among the ten ***Ulmus*** lines

The leaf blades on the ten *Ulmus* lines had different phenotypes (Table S1). The leaf shape of Lang was ovate, whereas the Jinye, Xingshan, and Duomai leaves were elliptic, and the leaves of the other six lines were obovate. Jinye had yellow leaves, whereas the other nine lines had green leaves. Among the *Ulmus* lines, Lieye was the only one whose leaf apex was 3–7 lobed. Finally, the leaf area of Lieye was the largest, whereas Zuiweng and Lang had the smallest leaves among the ten lines (*p* < 0.05).

**Fig. 1:**
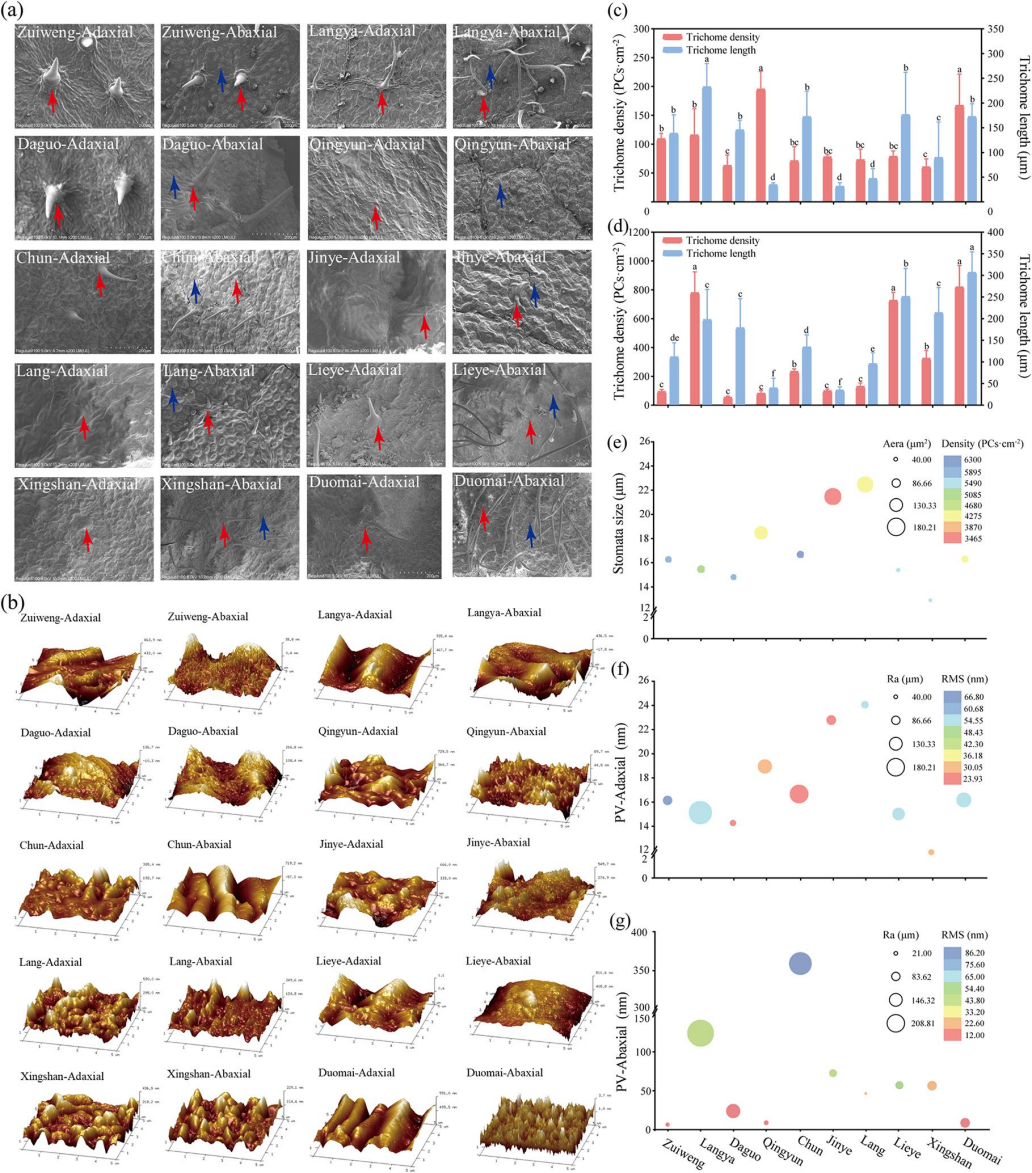
Leaf microstructure scans of ten *Ulmus* asexual lines. (**a**) Scanning electron microscopy images (200×); (**b**) atomic force microscopy images (5 × 5 μm); (**c** and **d**) quantification of the characteristics of adaxial and abaxial leaf trichomes, respectively; (**e**) quantification of the stomata properties; (**f** and **g**) quantification of the roughness of the adaxial and abaxial leaf surfaces, respectively. PV, peak and valley value; Ra, profile arithmetic average error; RMS, raw microroughness. The red and blue arrows in (**a**) indicate trichome and the stomata of the leaf surface, respectively. Results are presented as the mean ± SD (**c** and **d**) and mean (**e**, **f**, and **g**) of 15 independent experiments (*n* = 15). Different lowercase letters in (**c** and **d**) indicate significant differences (*p* < 0.05) among the ten lines of each index based on Tukey’s test

The two-dimensional images of the leaf surface of the ten *Ulmus* lines were obtained by SEM (Fig. 1a). The microstructures of both the adaxial and abaxial leaf surfaces varied greatly among the ten *Ulmus* lines. The adaxial trichomes were longer and curlier in Langya and more expanded at the base in Zuiweng than in the other lines. The density of the adaxial trichomes was higher in Qingyun and Duomai, whereas the density of the abaxial trichomes was higher in Langya, Lieye, and Duomai than in the other lines (*p* < 0.05). Finally, longer adaxial and abaxial trichomes were observed in Langya and Duomai, respectively, among the lines (*p* < 0.05; Fig. 1c and d). The stomatal size and area were larger in Lang and Jinye than in the other lines (*p* < 0.05), and their distribution was denser in Chun than in Duomai, Lang, Qingyun, and Jinye (*p* < 0.05), and less dense in Jinye than in Chun, Daguo, Lieye, Zuiweng, and Xingshan (*p* ≥ 0.05; Fig. 1e; Table S3).

The three-dimensional patterns of the leaf surface among the ten *Ulmus* lines were obtained by AFM (Fig. 1b). On the adaxial surface, Duomai exhibited a wavy conformation, and Lang had more microprotuberances than the other *Ulmus* lines. The abaxial surface of Lieye was flat, while that of Duomai was more rugged than those of the other lines. Besides, the profile arithmetic average error values of the adaxial and abaxial surfaces were higher (*p* < 0.05) in Langya than in the other lines, indicating that it had the greatest roughness (Fig. 1f and g; Table S3). The adaxial RMS value was lower (*p* < 0.05) in Daguo and Chun compared to that of the other eight lines, indicating a relative smooth adaxial leaf surface for those two lines(i.e., Daguo and Chun). In contrast, Chun exhibited a higher (*p* < 0.05) abaxial RMS value than all other lines, suggesting that it has the roughest abaxial leaf surface at the ultramicroscopic scale (*p* < 0.05). Concerning the adaxial PV values, Daguo had a lower (*p* < 0.05) PV value compared to the other lines, indicating a less undulating adaxial surface (*p* < 0.05), with no significant differences observed among the remaining lines (*p* ≥ 0.05). Additionally, Chun had the highest (*p* < 0.05) abaxial PV value among the ten lines (*p* < 0.05), further confirming the high roughness of its abaxial leaf surface (Fig. 1f and g; Table S3).

The ten *Ulmus* lines were classified into three categories using an unweighted pair group method with arithmetic mean clustering analysis of the above data (Fig. S3). The first category included only Chun, which was easily identified by its largely undulated abaxial surface and densely distributed stomata. Lieye, Langya, Zuiweng, Daguo, and Xingshan were included in the second category, of which Langya and Xingshan could be characterized by their dense abaxial trichomes, and Lieye, Zuiweng, and Daguo were identified by their sparsely distributed short abaxial trichomes. The third category consisted of the other four lines, of which Duomai, Qingyun, and Lang were identified by their sparsely distributed stomata and flat abaxial surfaces, and Jinye was identified by its sparsely distributed but extensive stomata (Fig. 1 and S3).

### The PM retention capacity on leaf surfaces varied among the ten ***Ulmus ***lines

**Fig. 2:**
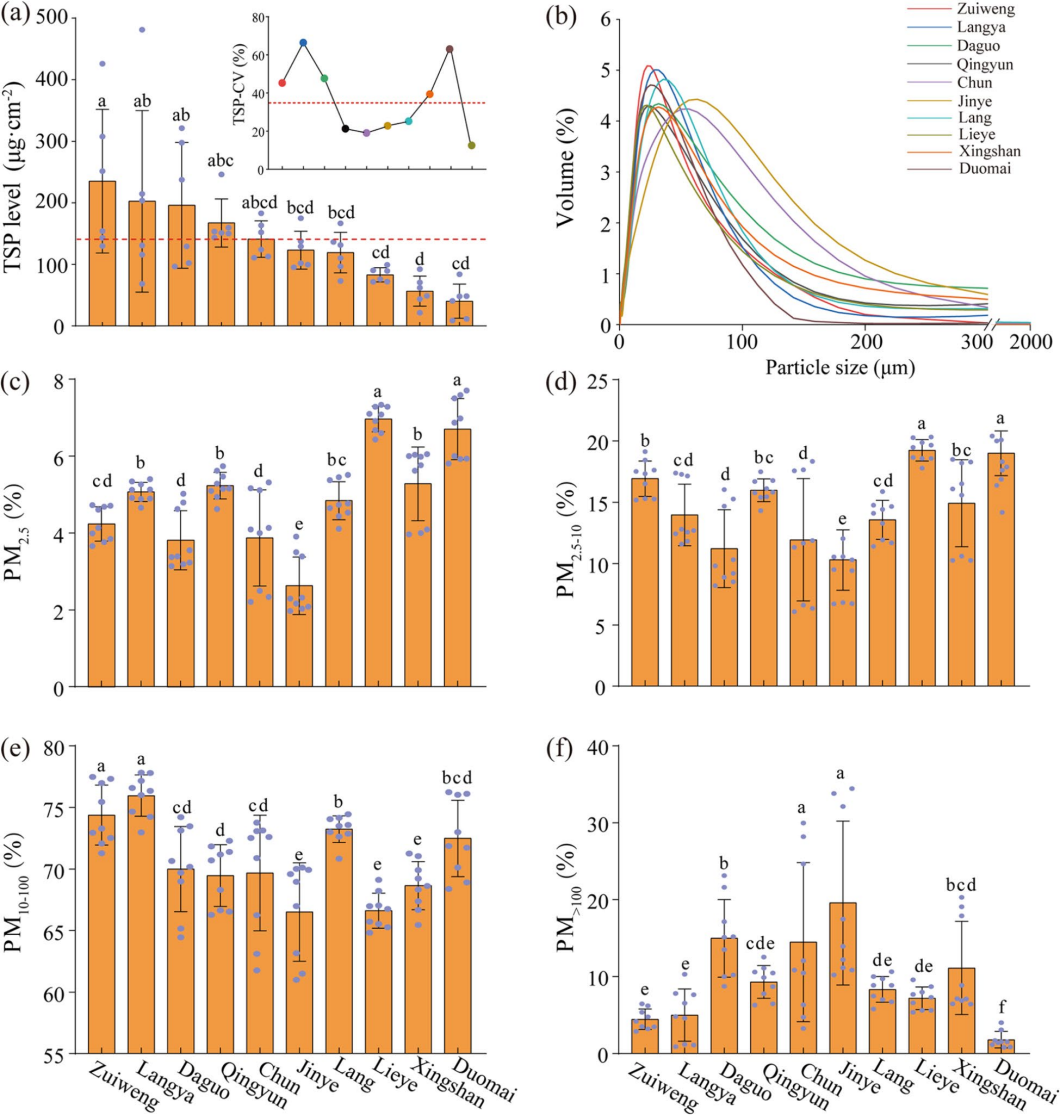
Leaf-retained total suspended particulate (TSP) and size distribution among ten *Ulmus* asexual lines. (**a**) TSP index values and the coefficient of variation; (**b**) size distribution curves; (**c**, **d**, **e**, and **f**) percentage particulate matter retention for different size classes. CV, coefficient of variation; PM, particulate matter; TSP, total suspended particulate. The red dashed lines in (**a**) represent the mean values. The TSP index values and its size distribution are presented as the mean ± SD of six (*n* = 6) and nine (*n* = 9) independent experiments, respectively. The dots in (**a**) and (**c**–**f**) represent the raw data. Different lowercase letters indicate significant differences (*p* < 0.05) among the ten lines based on Tukey’s test

The ten *Ulmus* lines could be divided into two groups of five lines each, based on whether they are above or below the mean TSP index values of the ten lines. The first group included Zuiweng, Langya, Daguo, Qingyun, and Chun; and the second group included Jinye, Lang, Duomai, Lieye, and Xingshan (Fig. 2a). Multiple comparisons revealed no significant differences (*p* ≥ 0.05) among the lines within the same group, except for Zuiweng, with a significantly higher (*p* < 0.05) TSP index value than the five lines in the second group. The TSP-CV is depicted as a measure of the relative stability of the foliar PM retention capacity among the ten *Ulmus* lines. Essentially, CV is a statistic that expresses the SD of a measured parameter as a percentage of the mean, offering insights into fluctuations in parameter performance across samples. Despite visual fluctuations in TSP-CV values among the ten *Ulmus* lines, Langya and Duomai exhibited the highest and lowest TSP-CV values, respectively, indicating a more variable TSP retention capacity in Langya and a more stable one in Duomai under the consistent tested conditions compared to those of the other lines (see the embedded figure in Fig. 2a).

The PM size distribution for all ten *Ulmus* lines witness a single peak mode (Fig. 2b): Chun and Jinye peaked at 56.37 and 63.25 μm, respectively, while the other eight lines peaked around 30 μm. Among the ten *Ulmus* lines, Duomai and Lieye possessed the highest PM_2.5_ and PM_2.5−10_ retention capacities (*p* < 0.05), whereas Daguo and Jinye had the lowest (*p* < 0.05; Fig. 2c and d). Zuiweng and Langya had the highest PM_10 − 100_ retention capacity, and Jinye, Lieye, and Xingshan had the lowest (*p* < 0.05; Fig. 2e); meanwhile, Jinye and Chun had the highest PM_> 100_ retention capacity, and Duomai had the lowest (*p* < 0.05; Fig. 2f).

### The phyllosphere microbial diversity patterns varied among the ten ***Ulmus ***lines

Sequencing quality control revealed that the mean values of tag efficiency obtained for bacteria and fungi were 94.387% and 94.099%, respectively. Besides, the rarefaction curves of all samples, whatever the bacterial and fungal communities, all reached a plateau (Fig. S4), indicating that the depth and coverage of the sequencing data were sufficient for the subsequent analyses. Thus, efficient tags with high sequence similarity were further clustered to form OTUs for microbial diversity, species annotation, and functional prediction analyses.

**Fig. 3:**
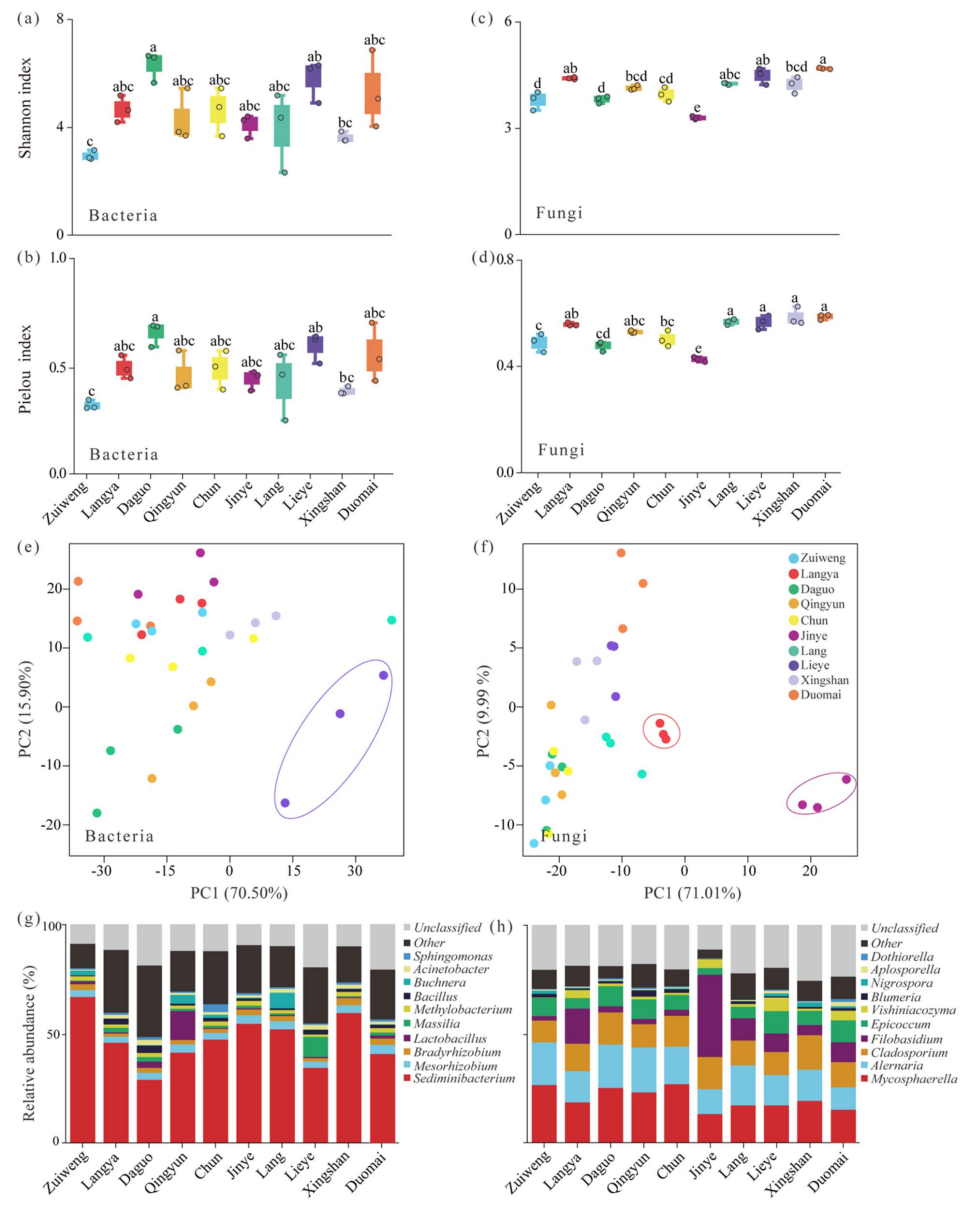
Phyllosphere microbial diversity among the ten *Ulmus* lines. (**a** − **d**) the Shannon and Pielou indices; (**e** and **f**) principal component analysis results; (**g** and **h**) histograms of the compositions of the top ten bacterial and fungal genera, respectively. PC, principal component

The alpha diversity (Shannon and Pielou indices) of the phyllosphere microbial communities were calculated at the OTU level in all the ten *Ulmus* lines (Fig. 3a–d). For the bacterial diversity, Daguo exhibited higher (*p* < 0.05) Shannon and Pielou index values compared to Xingshan and Zuiweng (*p* < 0.05; Fig. 3a and b). In contrast, Zuiweng had lower values (*p* < 0.05) in both two indices (i.e., the Shannon and Pielou indices) than Lieye (Fig. 3a and b). For the fungal community, Duomai had the highest (*p* < 0.05) Shannon index, followed in descending order by Zuiweng, Daguo, Qingyun, Chun, Jinye, Xingshan, and Jinye (Fig. 3c). The fungal Pielou index was higher in Duomai, Xingshan, Lieye, and Lang than in Zuiweng, Daguo, Chun, and Jinye, with Jinye having the lowest fungal Pielou value among the ten lines (*p* < 0.05; Fig. 3d).

The top two PCs explained the 86.40% and 81.00% variations in bacterial and fungal communities, respectively (Fig. 3e and f). PERMANOVA analysis results show that there were no significant differences (*p* > 0.05) in the bacterial and fungal diversity among the ten lines (Tables S4 and S5). Although most of the lines overlapped and could not be completely distinguished, Lieye (in terms of bacterial diversity) and Langya and Jinye (in terms of fungal diversity) could be distinguished from the other lines based on the distribution of sample scatters.

Next, the microbial species composition at the genus level in each of the ten *Ulmus* lines was investigated. The bacterial composition (Fig. 3g; Tables S6) was dominated by *Sediminibacterium*, while the fungi (Fig. 3h; Table S7) was dominated by *Mycosphaerella*, *Alternaria*, and *Cladosporium*. The relative abundances of the bacterial genus *Massilia* in Lieye and the fungal genus *Filobasidium* in Jinye were higher (*p* < 0.05) than in the other lines, respectively. These results demonstrate that the phyllosphere microbial diversity patterns varied among the ten *Ulmus* lines, of which Lieye and Jinye possessed unique phyllosphere bacterial and fungal microbial diversity patterns, respectively (Fig. 3e–h).

To provide a comprehensive view of the phyllosphere microbial community’s potential roles, a functional analysis of the microbial communities was conducted (Fig. S5). The results show that the functional classes of the bacterial communities (Fig. S5a) and their corresponding relative abundances were almost identical among the ten *Ulmus* lines. The relative abundances of “Amino acid metabolism” and “Carbohydrate metabolism” were particularly high. In contrast, the functional classes of the fungal communities (Fig. S5b) were not consistent among the ten lines. For example, the relative abundance “Symbiotroph” was higher (*p* < 0.05) in Jinye than in all of the other lines.

**Fig. 4:**
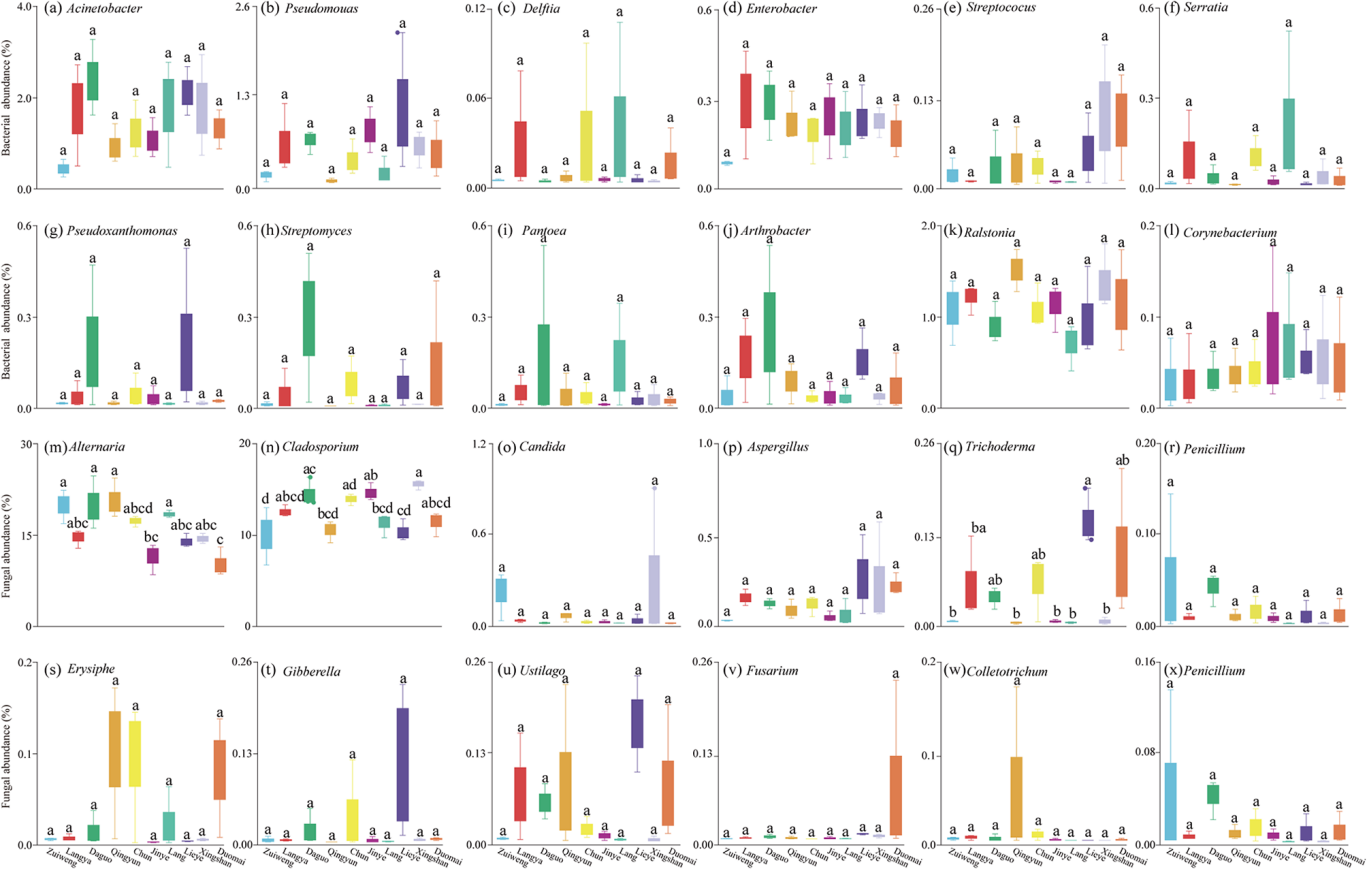
Quantification of the relative abundances of the 24 representative bacterial (**a**–**l**) and fungal (**m**–**x**) microorganisms. Different lowercase letters indicate significant differences (*p* < 0.05) based on Tukey’s test

The relative abundances of 24 representative microorganisms were further checked (Fig. 4). There were no differences (*p* < 0.05) in the leaf-retained relative abundance of bacterial microorganisms (Fig. 4a–l), but there were substantial differences among the fungal microorganisms (Fig. 4m–x) among the ten *Ulmus* lines. For example, the relative abundances of *Alternaria* retained on the leaf surface were higher (*p* < 0.05) in Zuiweng, Daguo, Qingyun, and Lang than in Jinye and Duomai.

### Leaf microstructures provided an ideal microhabitat for the retention of phyllosphere microorganisms carried by PM

**Fig. 5:**
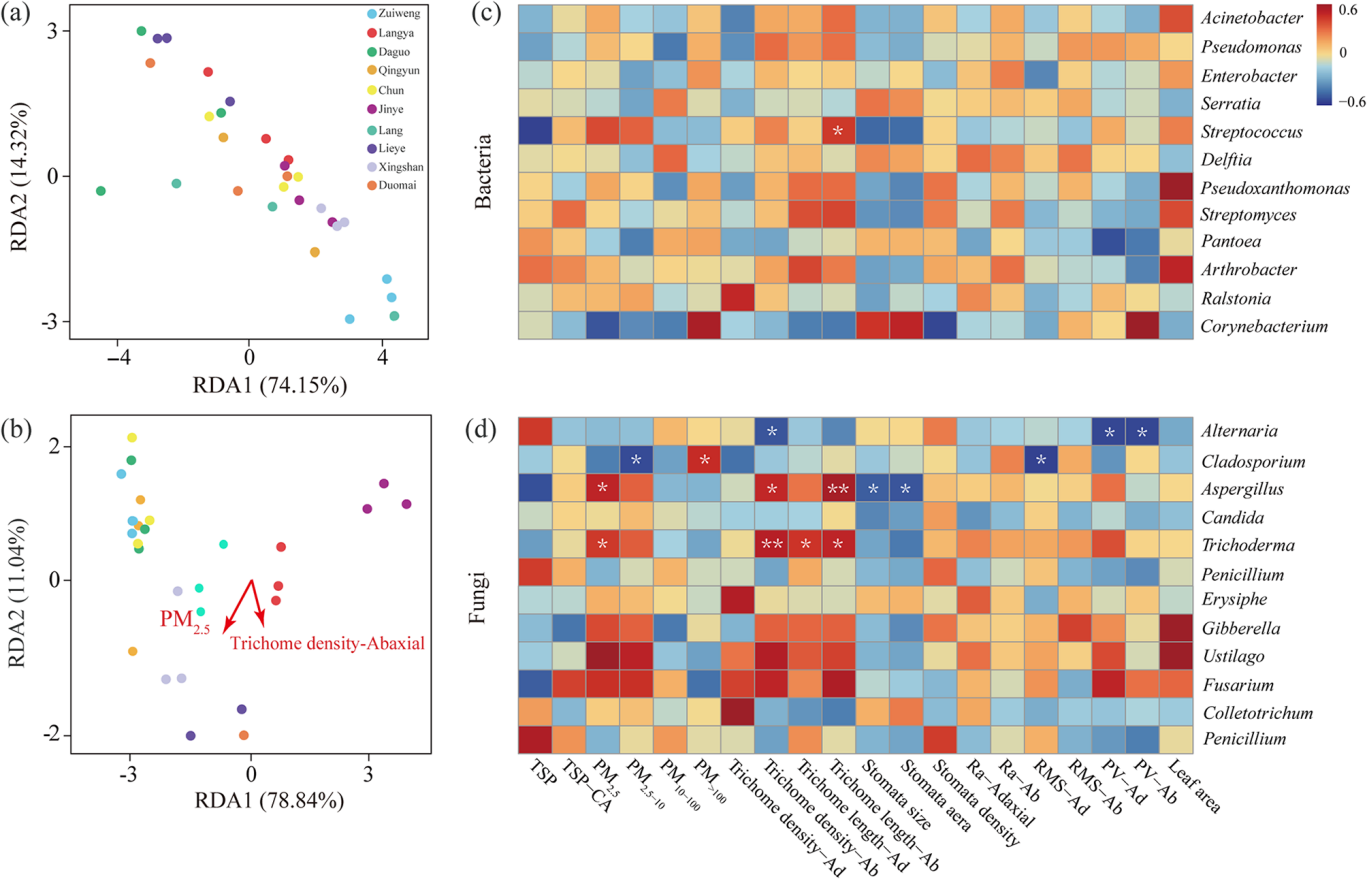
Effects of leaf surface microstructures and atmospheric particulate matter (PM) on phyllosphere microbial communities. (**a** and **b**) redundancy analysis diagrams of the bacterial and fungal microbial communities, respectively; (**c** and **d**) heat maps of the Pearson correlation coefficient values between PM factors and the microorganisms mentioned in Fig. 4; Ab, abaxial; Ad, adaxial; RDA, redundancy analysis; PM, particulate matter; PV, peak and valley value; Ra, profile arithmetic average error; RMS, raw roughness; TSP, total suspended particulate. The red arrows in (**a** and **b**) represent the environmental factors that significantly affected the microbial communities (*p* < 0.05), and the length of the arrow indicates the intensity of the effect. * and ** in (**c** and **d**) represent a significant correlation at *p* < 0.05 and *p* < 0.01, respectively. Experiments were all performed with three replicates (*n* = 3)

To better understand the effects of PM retained on a leaf surface and its microstructures on microbial communities, a RDA was performed by integrating the data shown in Figs. 1 and 2, and 3. The results show that Trichome density and PM_2.5_ retained on the abaxial surface had an effect (*p* < 0.05) on the fungal community, of which PM_2.5_ had the highest impact intensity (*R*^2^ = 0.711; Fig. 5b). However, no parameters had an impact (*p* > 0.05) on the bacterial community (Fig. 5a).

**Fig. 6:**
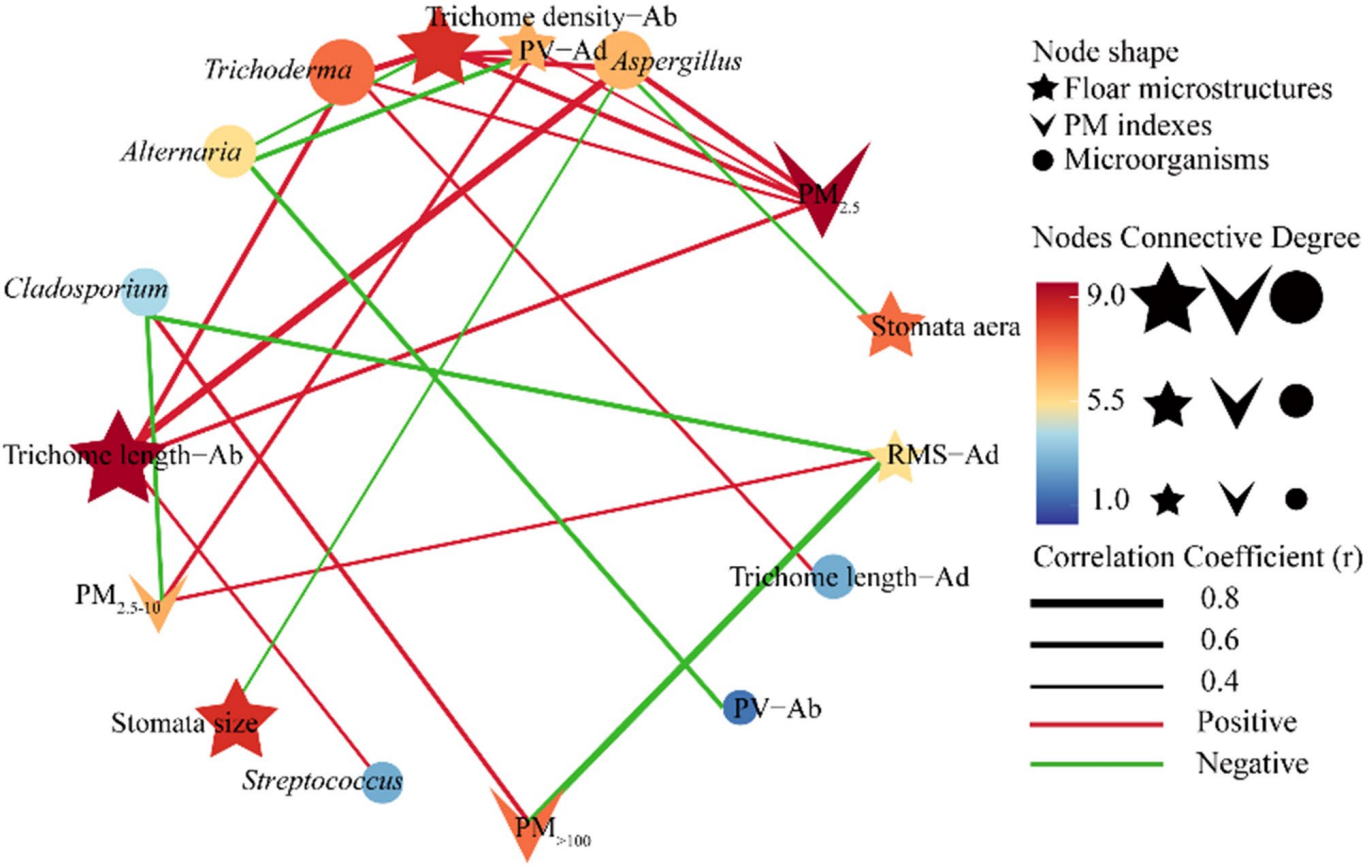
The network profile between PM, leaf microstructures, and microorganisms. PM, particulate matter; b, abaxial; Ad, adaxial; PV, peak and valley value; RMS, raw roughness

A correlation analysis was conducted to investigate whether leaf microstructures (Fig. 1) or PM (Fig. 2) could influence the relative abundance of retained microorganisms presented in Fig. 4. Figure 6c and d show that one bacterial (*Streptococcus*) and four fungal (*Alternaria*, *Cladosporium*, *Aspergillus*, and *Trichoderma*) microorganisms were correlated with at least one leaf microstructure or PM factor. For example, the relative abundances of *Trichoderma* and *Aspergillus* were positively correlated (*p* < 0.05) with PM_2.5_, abaxial trichome length, and abaxial trichome density, indicating that they could both interact with PM_2.5_ to form a PM_2.5_–microorganism complex, which could be easily captured by or attached to the dense, long trichomes on the leaf surface. The relative abundance of *Cladosporium* was correlated negatively (*p* < 0.05) with PM_2.5−10_ and adaxial RMS values but positively (*p* < 0.01) with PM_> 100_, suggesting that *Cladosporium* was not readily captured by leaf surface microstructures due to the larger size of the PM_> 100_–*Cladosporium* complex. Collectively, the above-mentioned correlation data (Fig. S6, 5c and d) between the PM, foliar microstructures, and the phyllosphere microorganisms were further condensed into a three-factor network profile (Fig. 6). A clear inherent connection was observed among the nodes within this profile. PM_2.5_ was the hub node within the network, exhibiting higher connectivity compared other nodes. In contrast, the abaxial PV value, adaxial trichome length, and the stomata size/area of the leaf surface each only connected with one microorganism (Fig. 6).

## Discussion

The assessment of leaf phenotypes is one of the most effective methods for identifying plant species [62]. Numerous studies have demonstrated that even within the same species, such as *Quercus variabilis*, *Quercus dentata*, and *Quercus rubra*, the leaf phenotypes of different asexual lines can be quite different [63, 64]. We found that although all experimental plants were grafted simultaneously and were cultivated under identical management strategies during the study, the micro- and ultramicro-structure of the leaf surfaces witnessed differences remarkably among the lines (Fig. 1 and Table S2), possibly due to structural differences or expression differences of the genes involved in regulating trichome and stomatal development, such as *R2R3*-*MYB* [65], *SBS* [66], *SPCH* [67], and *SCRM* [68], or to differences in the epidermal cell arrangement [69].

Trees are widely used in landscaping and as shelterbelts that can effectively retain atmospheric PM, although this ability varies among species [70]. The TSP index reflects the total amount of PM retained on the leaf surface, which can be used as an indicator to evaluate the PM retention capacity of plant species [71]. In this study, the average TSP values of the ten *Ulmus* lines (135.76 µg·cm^− 2^; Fig. 1) were much higher (by 59.62–231.44%) than most common woody plants under similar climatic conditions, including *Buxus megistophylla*, *Fraxinus pennsylvanica*, and *Sophora japonica* [5, 6, 72]. Besides, the TSP concentration at the study site was 167.36 ± 39.66 µg·m^− 3^ during the sampling period; in other words, a dollar coin-sized *Ulmus* leaf (with a double-sided area of 11.03 cm^2^) would be sufficient to remove about 8.95 µg·m^− 3^ of the PM. This suggests that the foliar PM retention capacity of these *Ulmus* lines was excellent. We found that PM size distribution for all ten *Ulmus* lines witnessed a single peak mode (Fig. 2b), which was similar to that of most woody plants, such as *Salix babylonica*, *Sophora japonica*, and *Ginkgo biloba* [71].

To better understand the reasons for the differences in the leaf PM retention capacity among the ten *Ulmus* lines, the leaf surface microscopic indices (Fig. 1) and the leaf retention PM indices (Fig. 2) were integrated for a correlation analysis (Fig. S6). We found that the PM_2.5_ retention capacities were positively correlated (*p* < 0.05) with the trichome density and length of the abaxial surface and the PV values of the adaxial surface. Moreover, PM_2.5−10_ retention capacities showed positive correlations (*p* < 0.05) with the RMS and the PV values of the adaxial surface. Therefore, long and dense trichomes contributed substantially to the PM_2.5_ retention capacity, and larger undulations on the leaf surface contributed substantially to the PM_10_ (D ≤ 10) retention capacity. The SEM images provided direct evidence to support this (Fig. S6b–d). Furthermore, the leaf PM_> 100_ retention capacity was negatively correlated (*p* < 0.01) with the RMS value of the adaxial surface, and no PM_> 100_ was found in any of the SEM images (Fig. S7), indicating that PM_> 100_ was not easily retained by the leaves. This well explains why the leaf PM retention curves among all ten lines peaked at < 100 μm (Fig. 2b). Moreover, this also suggests that the PM retention capacity of the ten *Ulmus* lines was dominated by leaf surface microstructures. These phenomena are possibly due to the long and dense trichomes and/or undulations on the leaf surface, which provide an increased surface area that can interact with airborne fine PM [73]. The increased surface area allows for a higher probability of PM adhesion through various physical forces, such as van der Waals forces or electrostatic interactions [74]. Besides, dense trichomes may create a more tortuous path for air movement across the leaf surface, causing turbulence that can enhance the deposition of fine PM [75]. In contrast, the retention of large PM by leaf surfaces appears to be less efficient, which may be attributed to the settling velocity of larger particles is higher due to their greater mass, which can lead to a greater propensity for these particles to bypass the leaf surface and settle directly to the ground [76, 77].

The leaf surfaces are inhabited by microbial communities [10], initiating intricate multipartite interactions involving the host plant, microorganisms, and the surrounding environment [14]. We found that even closely related *Ulmus* species/variety/cultivar differed significantly in their phyllosphere microbiome, in a homogeneous microbial environment (Figs. 3 and 4, and S5). Notably, Lieye and Jinye possessed unique phyllosphere bacterial and fungal microbial diversity patterns, respectively (Fig. 3). To further investigate the reason underlying this phenomenon, a correlation matrix (Table S8) was constructed based on their microbial diversity indices (Fig. 3), leaf microstructure indices (Fig. 1), and leaf-retained PM indices (Fig. 2). We found that the bacterial Shannon index of Lieye was negatively correlated (*p* < 0.05) with the stomata and trichome densities of its adaxial leaf surface, suggesting they contributed to the unique and high bacterial microbial diversity. The fungal Shannon index of Jinye was positively correlated (*p* < 0.05) with stomata density, but negatively correlated with the stomata area, indicating that the sparsely distributed but extensive stomata (Fig. 1e) were the main reason for the unique but low phyllosphere fungal microbial diversity in Jinye. This may stem from the influence of stomatal distribution and size on nutrient availability on the leaf surface, as they regulate gas exchange and water loss [78, 79]. Thus, a unique nutrient profile resulting from specific stomatal characteristics of plants might selectively promote or inhibit the growth of certain fungal groups.

The phyllosphere microorganisms may impact numerous processes and functions of the plants [14]. In this study, the predicted functions of the phyllosphere bacterial communities of the ten *Ulmus* lines’ phyllosphere microbiome were dominated by “Amino acid metabolism” and “Carbohydrate metabolism” (Fig. 5a). These two pathways are both crucial for the biosynthesis of essential biomolecules and energy production [80], suggesting that the phyllosphere bacterial community may affect the nutrient cycling and energy flow of the leaf. Moreover, the prevalence of “Pathotroph” and “Pathotroph-Saprotroph” functional classes among the fungal communities (Fig. 5b) indicates a potential for these fungi to engage in both pathogenic and saprophytic lifestyles. This dual role could have important implications for the health of the plants, as pathotrophs may contribute to disease resistance, while saprotrophs could aid in the decomposition of organic matter [81]. Notably, we also found that, unlike the bacterial communities, certain fungal functional classes, particularly “Symbiotroph”, varied among the ten lines, with significantly higher relative abundance observed in Jinye. This may be attributed to the ecological traits of the “Symbiotroph” class of fungi, potentially establishing a mutually beneficial symbiotic relationship with the plant [82]. For instance, similar to endophytes [83], phyllosphere microorganisms might provide the plant with antimicrobial substances or other beneficial compounds for defending pathogens, and in return, the plant provides the necessary resources for the fungi to thrive [84]. This may also indirectly reflect the potential higher tolerance of Jinye to biotic stress than the other lines, but it needs to be further verified.

Atmospheric PM conditions significantly impact the phyllosphere microbial community of many species [19, 85, 86]. Based on the RDA analysis (Fig. 5a and b), our results suggest that fungal microorganisms could be carried by the PM_2.5_ attached to the dense leaf trichomes. In contrast, bacteria were not easily carried by PM and were also difficult to capture by or attach to the leaf microstructures of the *Ulmus* lines. Based on a correlation profile (Fig. 5c and d), we found that several microorganisms (consisting of both bacteria and fungi) were correlated (*p* < 0.05) with PM factors and/or leaf microstructures, suggesting that the microstructures of the leaf surfaces of the ten *Ulmus* lines enable them to capture PM-borne microorganisms. For example, PM_2.5_ carried microorganisms, such as *Trichoderma* and *Aspergillus*; the microstructures of the leaf surface, especially the dense and long trichomes, provided an ideal microhabitat for the PM_2.5_-microorganism complexes (Fig. 5d). The two positive feedback loops (i.e., PM_2.5_ -abaxial trichome lengh-*Aspergillus* and PM_2.5_-abaxial trichome density- *Trichoderma*) within the correlation network profile also provides evidence to support this (Fig. 6). In contrast, within the profile, we also found two negative feedback loops (PM_2.5−10_/PM_> 100_-adaxial RMS values-*Cladosporium*), suggesting that PM in these size ranges is difficult to be retained by the microrough leaf adaxial surface, or even if captured, it is unable to promote/even inhibit the proliferation of specific microorganisms (Fig. 6). We further assessed the significance of the aforementioned four feedback loops through multiple regression analysis and confirmed their validity (*p* < 0.05) (Table S9). Overall, our data prove that plant foliar microstructures can create an ideal microhabitat for PM-borne microorganisms. On the other hand, whether and how these PM-borne microorganisms subsequently affect plant growth and development remains to be further explored.

## Conclusions

We demonstrate that the ten *Ulmus* lines investigated exhibited considerable PM retention capacities, with a mean TSP value of 135.76 µg·cm^− 2^. Variations in leaf surface microstructures, particularly the length and density of the trichome and the surface roughness, were the primary determinants of differential PM retention capacities among the lines. Long and dense trichomes substantially contributed to the retention of PM_2.5_, while larger undulations on the leaf surface enhanced the capture of PM_10_. Notably, these leaf microstructures provided ideal microhabitats for retaining PM-borne microorganisms, as evidenced by positive feedback loops between PM_2.5_, trichome characteristics, and the relative abundances of phyllosphere fungi like *Trichoderma* and *Aspergillus*. In contrast, bacterial communities were less impacted by PM. Our findings establish a three-factor network profile linking PM, leaf microstructures, and phyllosphere microbial communities, providing insights for further exploration into how different plants retain PM through foliar microstructures, thereby influencing their associated microbiomes.

### Electronic supplementary material

Below is the link to the electronic supplementary material.

Supplementary Material 1

Supplementary Material 2

## Data Availability

The raw sequencing data reported in this article have been publicly available under Genome Sequence Archivein in National Center for Bioinformation, China (https://ngdc.cncb.ac.cn/gsa; No. CRA016188 and CRA016189).
