# Supplementary material for: The relationship between atmospheric particulate matter, leaf surface microstructure, and the phyllosphere microbial diversity of Ulmus L
Source: BMC Plant Biol. 2024 Jun 17;24:566. doi: 10.1186/s12870-024-05232-z (PMC11181616; doi:10.1186/s12870-024-05232-z)
Supplement: Supplementary file 1 — Supplementary Material 1 [file 12870_2024_5232_MOESM1_ESM.docx]

**Table S1** Leaf characteristics of ten *Ulmus* asexual lines

| Abbr. | Shape | Apex | Color | Length (cm) | Width (cm) | Area (cm^2^) | Image |
| --- | --- | --- | --- | --- | --- | --- | --- |
| Zuiweng | Obovate | Acuminate, 0-lobed | Green | 5.41 ± 0.47d | 3.33 ± 0.46cd | 11.46 ± 1.45e | 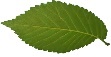 |
| Langya | Obovate | Shortly caudate, 0-lobed | Green | 9.67 ± 0.89c | 5.63 ± 0.45bc | 40.54 ± 3.27c | 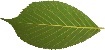 |
| Daguo | Obovate | Shortly caudate, 0-lobed | Green | 10.24 ± 1.02bc | 7.36 ± 0.67b | 51.37 ± 5.40bc | 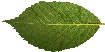 |
| Qingyun | Obovate | Acuminate, 0-lobed | Green | 8.06 ± 0.56cd | 3.51 ± 0.59cd | 21.45 ± 3.23d | 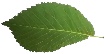 |
| Chun | Obovate | Caudate-acuminate, 0-lobed | Green | 8.01 ± 0.58cd | 3.56 ± 0.58cd | 21.18 ± 1.63d | 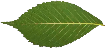 |
| Jinye | Elliptic | Acuminate, 0-lobed | Yellow | 5.82 ± 0.76d | 2.72 ± 0.23d | 9.99 ± 0.49e | 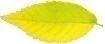 |
| Lang | Ovate | Obtuse, 0-lobed | Green | 5.26 ± 0.66d | 2.74 ± 0.29d | 12.28 ± 0.46e | 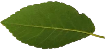 |
| Lieye | Obovate | Caudate-acuminate, 3-7lobed | Green | 15.99 ± 0.67a | 9.41 ± 0.58a | 108.45 ± 7.03a | 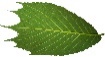 |
| Xingshan | Elliptic | Acuminate-caudate, 0-lobed | Green | 8.71 ± 0.55cd | 4.25 ± 0.45c | 28.62 ± 2.70d | 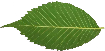 |
| Duomai | Elliptic | Cuspidate, 0-lobed | Green | 11.34 ± 1.33b | 5.79 ± 0.23bc | 62.23 ± 11.23b | 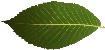 |

Notes: The data were obtained by measurement of 30 independent leaves (n = 30) within each of the ten *Ulmus* lines and presented as the mean ± SD. Different lowercase letters indicate significant differences (*p* < 0.05) based on the Tukey's test.

**Table S2** Information of the 24 selected representative microorganisms

| No. | Microbial type | Representative microorganisms | Reference | Author | Year |
| --- | --- | --- | --- | --- | --- |
| 1 | Bacteria | *Acinetobacter* | Characterization of plant-growth-promoting traits of *Acinetobacter* species isolated from rhizosphere of *Pennisetum glaucum* | Rokhbakhsh-Zamin et al. | 2011 |
| 2 | Bacteria | *Pseudomonas* | Plant perceptions of plant growth-promoting *Pseudomonas* | Preston et al. | 2004 |
| 3 | Bacteria | *Enterobacter* | *Enterobacter*: role in plant growth promotion | Jha et al. | 2011 |
| 4 | Bacteria | *Serratia* | Using *Serratia* plymuthica to control fungal pathogens of plants | De Vleesschauwer et al. | 2003 |
| 5 | Bacteria | Streptococcus | The antibacterial activity of plant extracts containing polyphenols against *Streptococcus* mutans | Smullen et al. | 2007 |
| 6 | Bacteria | *Delfia* | Degradation of acetaminophen by *Delftia* *tsuruhatensis* and *Pseudomonas* *aeruginosa* in a membrane bioreactor | De Gusseme et al. | 2011 |
| 7 | Bacteria | *Pseudoxanthomonas* | The versatility and adaptation of bacteria from the genus *Stenotrophomonas* | Ryan et al. | 2009 |
| 8 | Bacteria | *Streptomyces* | The role of *Streptomyces* species in controlling plant diseases: a comprehensive review | Quwaie et al. | 2024 |
| 9 | Bacteria | *Pantoea* | *Pantoea*: insights into a highly versatile and diverse genus within the Enterobacteriaceae | Alyssa et al. | 2015 |
| 10 | Bacteria | *Arthrobacter* | An isolated *Arthrobacter*sp. Enhances rice (*Oryza sativa* L.) plant growth | Geeta et al. | 2022 |
| 11 | Bacteria | *Ralstonia* | Ralstonia solanacearum – A soil borne hidden enemy of plants: Research development in management strategies, their action mechanism and challenges | Wang et al. | 2023 |
| 12 | Bacteria | *Corynebacterium* | Growth enhancement and root colonization of rice seedlings by *Rhizobium* and *Corynebacterium* spp. | Naher et al. | 2009 |
| 13 | Fungi | *Alternaria* | Could *Alternaria solani* IA300 be a plant growth-promoting fungus? | Mauricio-Castillo et al. | 2020 |
| 14 | Fungi | *Cladosporium* | *Cladosporium* sp. isolate as fungal plant growth promoting agent | Răut et al. | 2021 |
| 15 | Fungi | *Aspergillus* | Applications of *Aspergillus* in plant growth promotion | Hung et al. | 2016 |
| 16 | Fungi | *Candida* | Plant-growth promoting *Candida* sp. AVGB4 with capability of 4-nitroaniline biodegradation under drought stress | Silambarasan et al. | 2017 |
| 17 | Fungi | *Tiichoderma* | Applications of *Trichoderma* in plant growth promotion | Stewart et al. | 2014 |
| 18 | Fungi | *Penicillium* | Characterization of plant growth-promoting traits of *Penicillium* species against the effects of high soil salinity and root disease | Radhakrishnan et al. | 2014 |
| 19 | Fungi | *Erysiphe* | First comprehensive phylogenetic analysis of the genus *Erysiphe* (Erysiphales, Erysiphaceae) I. The *Microsphaera* lineage | Takamatsu et al. | 2015 |
| 20 | Fungi | *Gibberella* | Natural occurrence of perithecia of *Gibberella coronicola* on wheat plants with crown rot in Australia | Summerell et al. | 2001 |
| 21 | Fungi | *Ustilago* | Fungal development of the plant pathogen *Ustilago* *maydis* | Vollmeister et al. | 2012 |
| 22 | Fungi | *Fusarium* | *Fusarium* diseases of cultivated plants, control, diagnosis, and molecular and genetic studies | Arie and Tsutomu | 2019 |
| 23 | Fungi | *Colletotrichum* | Life styles of *Colletotrichum* species and implications for plant biosecurity | De Silva et al. | 2017 |
| 24 | Fungi | *Penicillium* | Plant growth promotion and Penicillium citrinum | Khan et al. | 2008 |

**Table S3** Characteristics of the leaf microstructures of ten *Ulmus* asexual lines

|  | Stomata | | | Ra | | RMS | | PV | |
| --- | --- | --- | --- | --- | --- | --- | --- | --- | --- |
|  | Density (PCs·cm^-2^) | Size (μm) | Area (μm^2^) | Ad^*^ (μm) | Ab (μm) | Ad (nm) | Ab (nm) | Ad (nm) | Ab (nm) |
| Zuiweng | 5794.28 ± 434.08ab | 16.32 ± 2.81bc | 68.91 ± 15.82cd | 95.20 ± 30.74bc | 32.10 ± 16.79c | 66.62 ± 83.63a | 2.39 ± 0.79c | 126.96 ± 73.60abc | 7.81 ± 5.14b |
| Langya | 4925.14 ± 1151.44abc | 15.6 ± 1.73cd | 80.99 ± 26.26c | 240.00 ± 155.51a | 2090.00 ± 149.37a | 50.66 ± 25.66ab | 33.73 ± 16.37bc | 180.24 ± 88.77ab | 124.80 ± 88.68b |
| Daguo | 5545.95 ± 929.15ab | 14.99 ± 2.13cd | 61.90 ± 27.16cd | 65.10 ± 47.73c | 109.70 ± 32.81bc | 17.98 ± 14.01c | 6.19 ± 5.38c | 30.64 ± 16.85d | 25.61 ± 12.87b |
| Qingyun | 3973.22 ± 756.39bc | 18.31 ± 1.77b | 138.49 ± 42.45b | 146.20 ± 102.96b | 36.10 ± 14.94c | 24.10 ± 14.41ab | 5.99 ± 2.42c | 98.32 ± 61.38abc | 10.69 ± 7.63b |
| Chun | 6290.93 ± 1175.00a | 16.69 ± 2.16bc | 75.04 ± 17.44c | 191.5 ± 94.66b | 176.30 ± 132.32b | 18.76 ± 15.65c | 86.10 ± 70.76a | 86.93 ± 135.43abc | 359.10 ± 287.59a |
| Jinye | 3062.69 ± 304.14c | 21.03 ± 2.06a | 173.41 ± 39a | 98.60 ± 12.79bc | 59.60 ± 24.12c | 21.99 ± 11.68ab | 35.28 ± 23.52bc | 130.94 ± 121.82abc | 73.83 ± 92.19b |
| Lang | 4262.93 ± 854.23bc | 21.94 ± 2.12a | 165.39 ± 33.35a | 79.80 ± 23.13bc | 21.20 ± 30.41c | 51.01 ± 36.12ab | 19.24 ± 8.16bc | 110.79 ± 46.31abc | 48.04 ± 38.85b |
| Lieye | 5214.85 ± 154.86ab | 15.53 ± 2.6cd | 43.25 ± 10.17d | 128.40 ± 97.5bc | 61.90 ± 40.41c | 53.13 ± 18.80ab | 46.00 ± 21.39b | 188.04 ± 75.43ab | 58.53 ± 47.05b |
| Xingshan | 5256.24 ± 769.86ab | 13.27 ± 1.3d | 40.11 ± 8.10d | 60.10 ± 34.80c | 72.90 ± 48.27c | 27.07 ± 23.45ab | 15.68 ± 16.30bc | 112.55 ± 58.28abc | 57.90 ± 38.98b |
| Duomai | 4056.00 ± 769.86bc | 16.35 ± 1.63bc | 71.64 ± 14.53cd | 153.00 ± 142.42bc | 74.90 ± 109.71c | 50.40 ± 37.57ab | 1.49 ± 3.05d | 213.39 ± 139.17a | 0.43 ± 0.43b |

Notes: Ad, adaxial; Ab, abaxial. Results are presented as mean ± SD of fifteen independent experiments (n = 15). Different lowercase letters indicate significant differences among lines (*p* < 0.05) based on Tukey's test.

**Table S4** PERMANOVA test of the differences in bacterial community structures

| Group | Zuiweng | Langya | Daguo | Chun | Jinye | Lang | Duomai | Lieye | Xingshan |
| --- | --- | --- | --- | --- | --- | --- | --- | --- | --- |
| Qingyun | 0.155 | 0.155 | 0.155 | 0.243 | 0.155 | 0.500 | 0.155 | 0.155 | 0.155 |
| Zuiweng |  | 0.155 | 0.155 | 0.155 | 0.155 | 0.243 | 0.155 | 0.155 | 0.155 |
| Langya |  |  | 0.155 | 0.329 | 0.155 | 0.329 | 0.155 | 0.155 | 0.155 |
| Daguo |  |  |  | 0.243 | 0.155 | 0.243 | 0.155 | 0.155 | 0.155 |
| Chunmei |  |  |  |  | 0.329 | 0.500 | 0.243 | 0.155 | 0.329 |
| Jinye |  |  |  |  |  | 0.429 | 0.243 | 0.155 | 0.155 |
| Lang |  |  |  |  |  |  | 0.243 | 0.243 | 0.500 |
| Duomai |  |  |  |  |  |  |  | 0.155 | 0.155 |
| Lieye |  |  |  |  |  |  |  |  | 0.155 |

Note: The data are *P*-value matrix between ten *Ulmus* lines based on permutational analysis of variance (PERMANOVA) analysis.

**Table S5** PERMANOVA test of the differences in fungal community structures

| Group | Zuiweng | Langya | Daguo | Chun | Jinye | Lang | Duomai | Lieye | Xingshan |
| --- | --- | --- | --- | --- | --- | --- | --- | --- | --- |
| Qingyun | 0.102 | 0.102 | 0.102 | 0.102 | 0.102 | 0.102 | 0.102 | 0.102 | 0.102 |
| Zuiweng |  | 0.102 | 0.102 | 0.102 | 0.102 | 0.102 | 0.102 | 0.102 | 0.102 |
| Langya |  |  | 0.102 | 0.102 | 0.102 | 0.102 | 0.102 | 0.102 | 0.102 |
| Daguo |  |  |  | 0.400 | 0.102 | 0.102 | 0.102 | 0.102 | 0.102 |
| Chunmei |  |  |  |  | 0.102 | 0.102 | 0.102 | 0.102 | 0.102 |
| Jinye |  |  |  |  |  | 0.102 | 0.102 | 0.102 | 0.102 |
| Lang |  |  |  |  |  |  | 0.102 | 0.102 | 0.102 |
| Duomai |  |  |  |  |  |  |  | 0.102 | 0.102 |
| Lieye |  |  |  |  |  |  |  |  | 0.102 |

Note: The data are *P*-value matrix between ten *Ulmus* lines based on permutational analysis of variance (PERMANOVA) analysis.

**Table S6** Relative abundance values of the top five bacterial genera

| Genus | Zuiweng | Langya | Qingyun | Daguo | Chun | Jinye | Lang | Lieye | Xingshan | Duomai |
| --- | --- | --- | --- | --- | --- | --- | --- | --- | --- | --- |
| *Sediminibacterium* | 47.21 ± 7.47a | 23.42 ± 5.26a | 23.32 ± 6.11a | 14.2 ± 6.95b | 24.93 ± 9.36a | 26.56 ± 6.03a | 32.2 ± 23.63a | 20.27 ± 5.55a | 35.84 ± 3.62a | 13.91 ± 6.15b |
| *Mesorhizobium* | 2.25 ± 0.72a | 1.4 ± 0.33a | 2.15 ± 0.69a | 1.51 ± 0.26a | 1.53 ± 0.20a | 1.93 ± 0.29a | 1.97 ± 0.47a | 1.76 ± 1.11a | 2.17 ± 0.31a | 1.45 ± 0.58a |
| *Bradyrhizobium* | 1.91 ± 0.72a | 0.69 ± 0.16a | 1.11 ± 0.57a | 1.05 ± 0.15a | 0.88 ± 0.26a | 1.24 ± 0.39a | 1.28 ± 0.58a | 0.87 ± 0.3a | 1.93 ± 0.71a | 1.01 ± 0.69a |
| *Massilia* | 0.23 ± 0.17b | 1.06 ± 0.43b | 0.61 ± 0.29b | 0.96 ± 0.19b | 0.51 ± 0.08b | 0.52 ± 0.40b | 0.39 ± 0.20b | 5.39 ± 0.57a | 0.28 ± 0.10b | 0.43 ± 0.08b |
| *Methylobacterium* | 1.43 ± 0.71a | 0.74 ± 0.18a | 0.84 ± 0.22a | 0.88 ± 0.10a | 1.04 ± 0.55a | 1.03 ± 0.25a | 0.78 ± 0.26a | 0.62 ± 0.18a | 1.16 ± 0.28a | 0.67 ± 0.16a |

Notes: The data are relative abundance of the top five bacterial genera among ten *Ulmus* lines presented as mean ± SD of three (n = 3) independent experiments. Different lowercase letters indicate significant differences (*p* < 0.05) among the ten lines of each bacterial genus based on Tukey's test.

**Table S7** Relative abundance values of the top five fungal genera

|  | Zuiweng | Langya | Qingyun | Daguo | Chun | Jinye | Lang | Lieye | Xingshan | Duomai |
| --- | --- | --- | --- | --- | --- | --- | --- | --- | --- | --- |
| *Mycosphaerella* | 26.40 ± 1.97a | 18.46 ± 2.27bc | 23.01 ± 2.36ab | 25.07 ± 1.29a | 26.80 ± 2.52a | 13.10 ± 0.29d | 17.04 ± 0.70cd | 17.07 ± 0.73cd | 19.12 ± 1.91bc | 15.07 ± 1.30cd |
| *Alternaria* | 19.60 ± 2.75a | 14.31 ± 1.50abc | 20.66 ± 3.25a | 19.92 ± 4.35a | 17.25 ± 0.88ab | 11.35 ± 2.53bc | 18.4 ± 0.59a | 13.95 ± 1.13abc | 14.38 ± 0.82abc | 10.28 ± 2.40c |
| *Cladosporium* | 10.01 ± 3.18d | 12.62 ± 0.65abcd | 10.66 ± 1.2bcd | 14.73 ± 1.58abc | 14.12 ± 0.64abcd | 14.85 ± 0.97ab | 11.39 ± 1.33bcd | 10.53 ± 1.24cd | 15.76 ± 0.54a | 11.55 ± 1.37abcd |
| *Filobasidium* | 2.04 ± 0.39e | 16.11 ± 0.82b | 2.39 ± 1.15e | 2.86 ± 0.42e | 2.82 ± 0.93e | 37.76 ± 2.82a | 10.23 ± 2.13cd | 8.51 ± 0.59cd | 4.68 ± 1.91de | 9.17 ± 0.78cd |
| *Epicoccum* | 8.44 ± 1.81ab | 4.82 ± 0.67ab | 9.02 ± 3.23ab | 9.20 ± 0.78a | 6.78 ± 2.26ab | 3.07 ± 1.13b | 5.28 ± 1.48ab | 10.28 ± 1.75a | 6.53 ± 1.24ab | 10.02 ± 3.86a |

Notes: The data are relative abundance of the top five fungal genera among ten *Ulmus* lines presented as mean ± SD of three (n = 3) independent experiments. Different lowercase letters indicate significant differences (*p* < 0.05) among the ten lines of each bacterial genus based on Tukey's test.

**Table S8** Correlation matrix of the phyllosphere microbial diversities, leaf microstructures, and leaf-retained PM factors of Lieye and Jinye

|  |  |  | Leaf area | Stomata | | | Ra | | RMS | | PV | | TSP | PM | | | | Trichome density | | Trichome length | |
| --- | --- | --- | --- | --- | --- | --- | --- | --- | --- | --- | --- | --- | --- | --- | --- | --- | --- | --- | --- | --- | --- |
|  |  |  |  | Density | Size | area | Ad* | Ab | Ad | Ab | Ad | Ab |  | 2.5 | 10 | 100 | >100 | Ad | Ab | Ad | Ab |
| Lieye | Bacterial Shannon index | *r* | -0.418 | -0.997* | -0.034 | 0.05 | 0.827 | 0.458 | -0.873 | -0.825 | -0.951 | 0.823 | -0.996 | 0.98 | 0.687 | 0.93 | -0.972 | -0.875* | 0.88 | -0.969 | -0.129 |
|  |  | *p* | 0.725 | 0.047 | 0.978 | 0.968 | 0.38 | 0.697 | 0.324 | 0.383 | 0.201 | 0.385 | 0.055 | 0.127 | 0.129 | 0.239 | 0.151 | 0.047 | 0.315 | 0.159 | 0.917 |
|  | Bacterial Pielou index | *r* | -0.441 | -0.995 | -0.06 | 0.024 | 0.812 | 0.435 | -0.861 | -0.81 | -0.958 | 0.808 | -0.994 | 0.985 | 0.995 | 0.939 | -0.978 | -0.995 | 0.892 | -0.975 | -0.104 |
|  |  | *p* | 0.709 | 0.063 | 0.962 | 0.985 | 0.397 | 0.713 | 0.34 | 0.399 | 0.185 | 0.401 | 0.071 | 0.111 | 0.065 | 0.223 | 0.134 | 0.063 | 0.298 | 0.143 | 0.934 |
| Jinye | Fungal Shannon index | *r* | -0.948 | 0.913* | -0.761 | -0.886* | 0.28 | 0.847 | -0.916 | -0.753 | 0.993 | -0.843 | -0.308 | 0.87 | 0.314 | 0.189 | -0.298 | -0.951 | -0.668 | -0.334 | -0.84 |
|  |  | *p* | 0.207 | 0.011 | 0.449 | 0.013 | 0.82 | 0.357 | 0.262 | 0.457 | 0.077 | 0.361 | 0.801 | 0.328 | 0.797 | 0.879 | 0.807 | 0.201 | 0.534 | 0.783 | 0.365 |
|  | Fungal Pielou index | *r* | -0.589 | 0.961 | -0.917 | -0.962 | -0.013 | 0.965 | -0.993 | -0.528 | 0.914 | -0.963 | -0.572 | 0.688 | 0.578 | 0.468 | -0.564 | -0.818 | -0.421 | -0.044 | -0.645 |
|  |  | *p* | 0.118 | 0.177 | 0.261 | 0.176 | 0.991 | 0.168 | 0.073 | 0.646 | 0.266 | 0.173 | 0.612 | 0.517 | 0.608 | 0.69 | 0.618 | 0.39 | 0.723 | 0.972 | 0.554 |

Notes: Ab, abaxial; Ad, adaxial; PM, particulate matter; PV, peak and valley value; Ra, profile arithmetic average error; RMS, raw microroughness. *r* represents the Pearson correlation coefficient. Asterisks represent significant correlation (*p* < 0.05) between alpha diversity index (i.e., Shannon and/or Pielou) with leaf microstructures.

**Table S9** Model formula for phyllosphere microorganisms, PM, and foliar microstructures

| Implicit variable (*y*) | Independent variable | | Model formula | *R*^2^ | *P*-value |
| --- | --- | --- | --- | --- | --- |
|  | *x*_1_ | *x*_2_ |  |  |  |
| *Aspergillus* | PM_2.5_ | Abaxial trichome lengh | *y* = -0.069 + 0.019 *x*_1_ + 0.001 *x*_2_ | 0.772 | 0.006 |
| *Trichoderma* | PM_2.5_ | Abaxial trichome density | *y* = -0.027 + 0.007 *x*_1_ + 0.002 *x*_2_ | 0.638 | 0.029 |
| *Cladosporium* | PM_2.5-10_ | Adaxial RMS values | *y* = 18.785-0.269 *x*_1_ - 0.057 *x*_2_ | 0.633 | 0.030 |
| *Cladosporium* | PM_>100_ | Adaxial RMS values | *y* = 13.378 + 0.133 *x*_1_ - 0.053 *x*_2_ | 0.591 | 0.044 |

Notes: PM denotes particulate matter. *R*^2^ indicates the degree of fit for the model. A *P*-value less than 0.05 indicates a significant fit.
