# Supplementary material for: The relationship between atmospheric particulate matter, leaf surface microstructure, and the phyllosphere microbial diversity of Ulmus L
Source: BMC Plant Biol. 2024 Jun 17;24:566. doi: 10.1186/s12870-024-05232-z (PMC11181616; doi:10.1186/s12870-024-05232-z)
Supplement: Supplementary file 2 — Supplementary Material 2 [file 12870_2024_5232_MOESM2_ESM.docx]

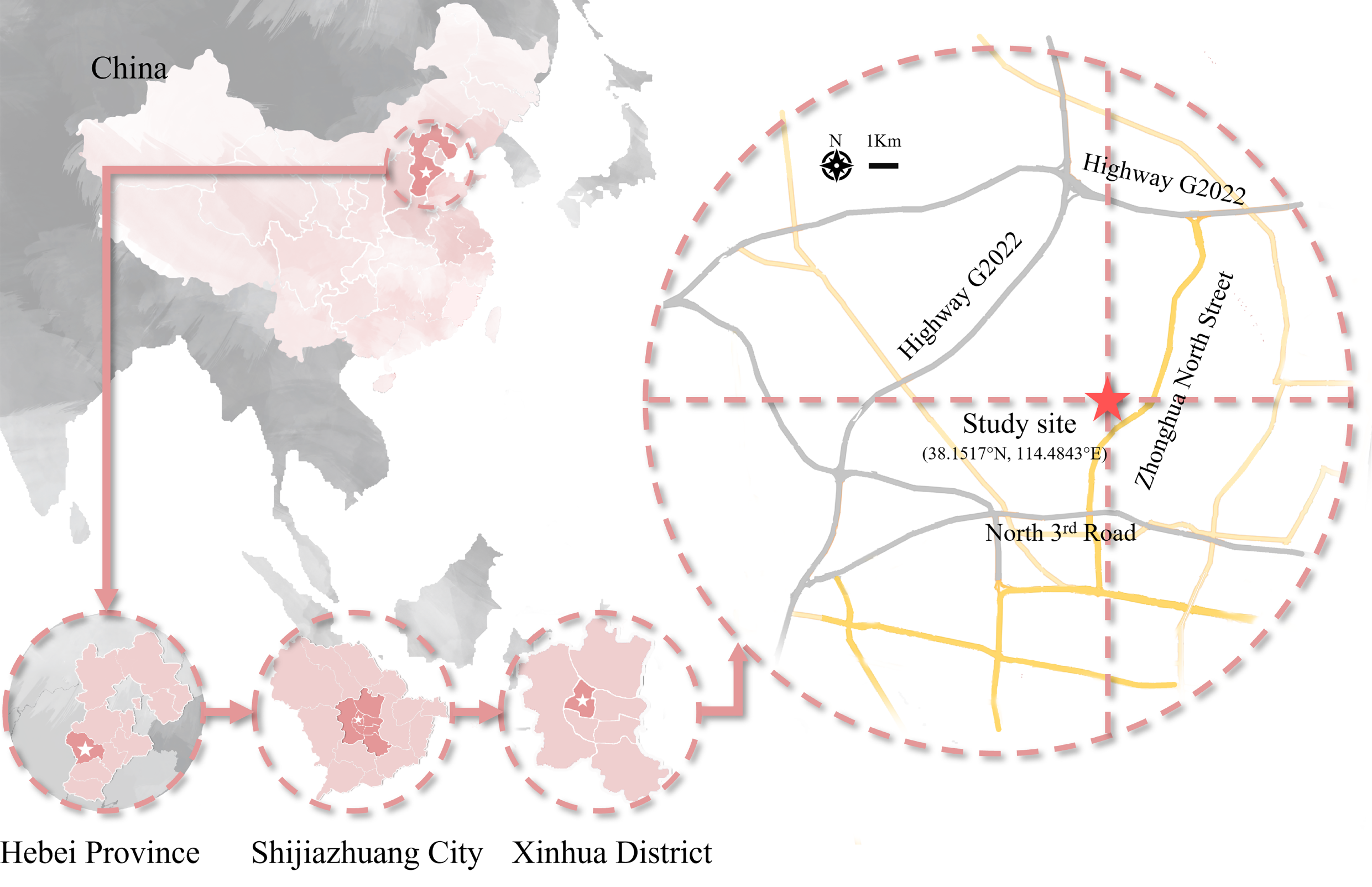


**Fig. S1** Location of the study site in Xinhua District, Shijiazhuang, Hebei Province, China.


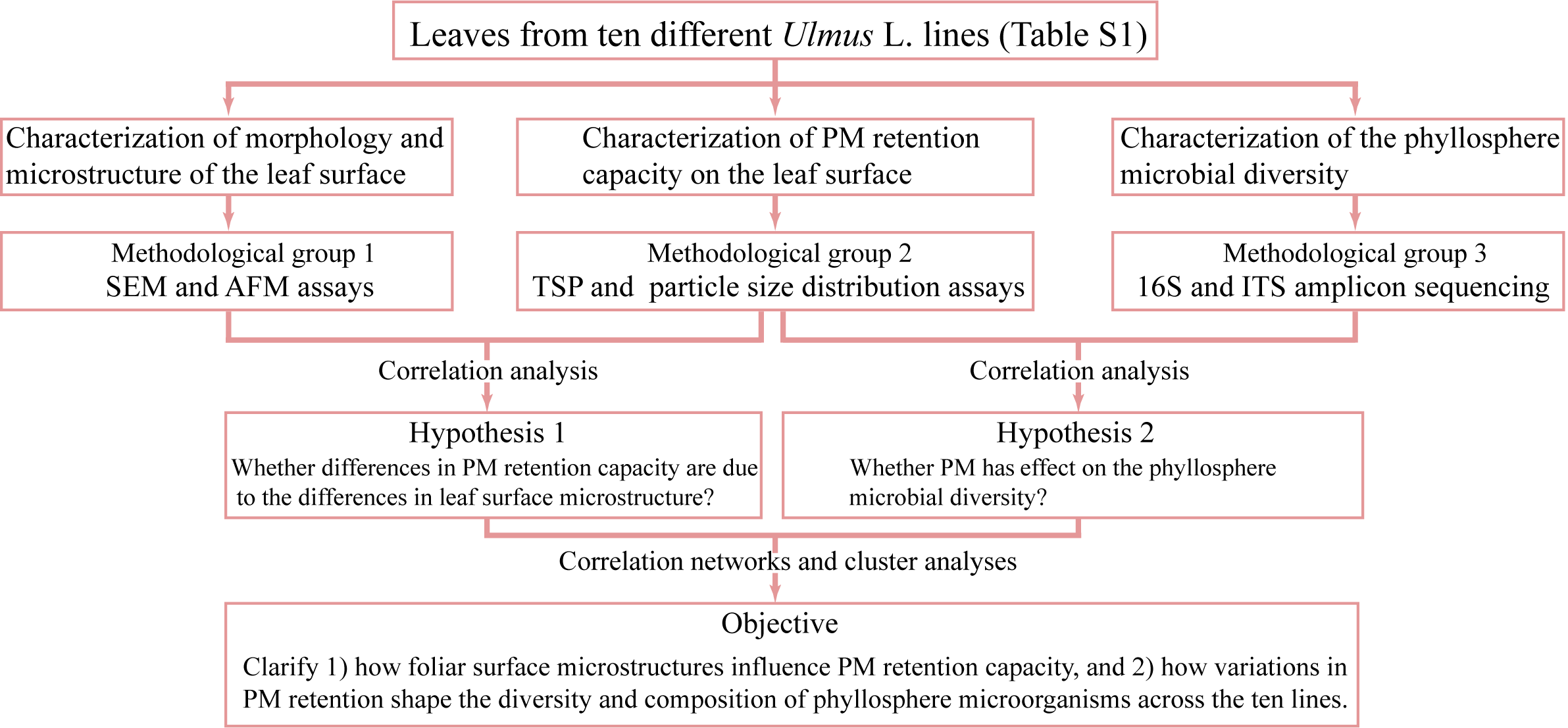


**Fig. S2** Flowchart of the experimental processes.


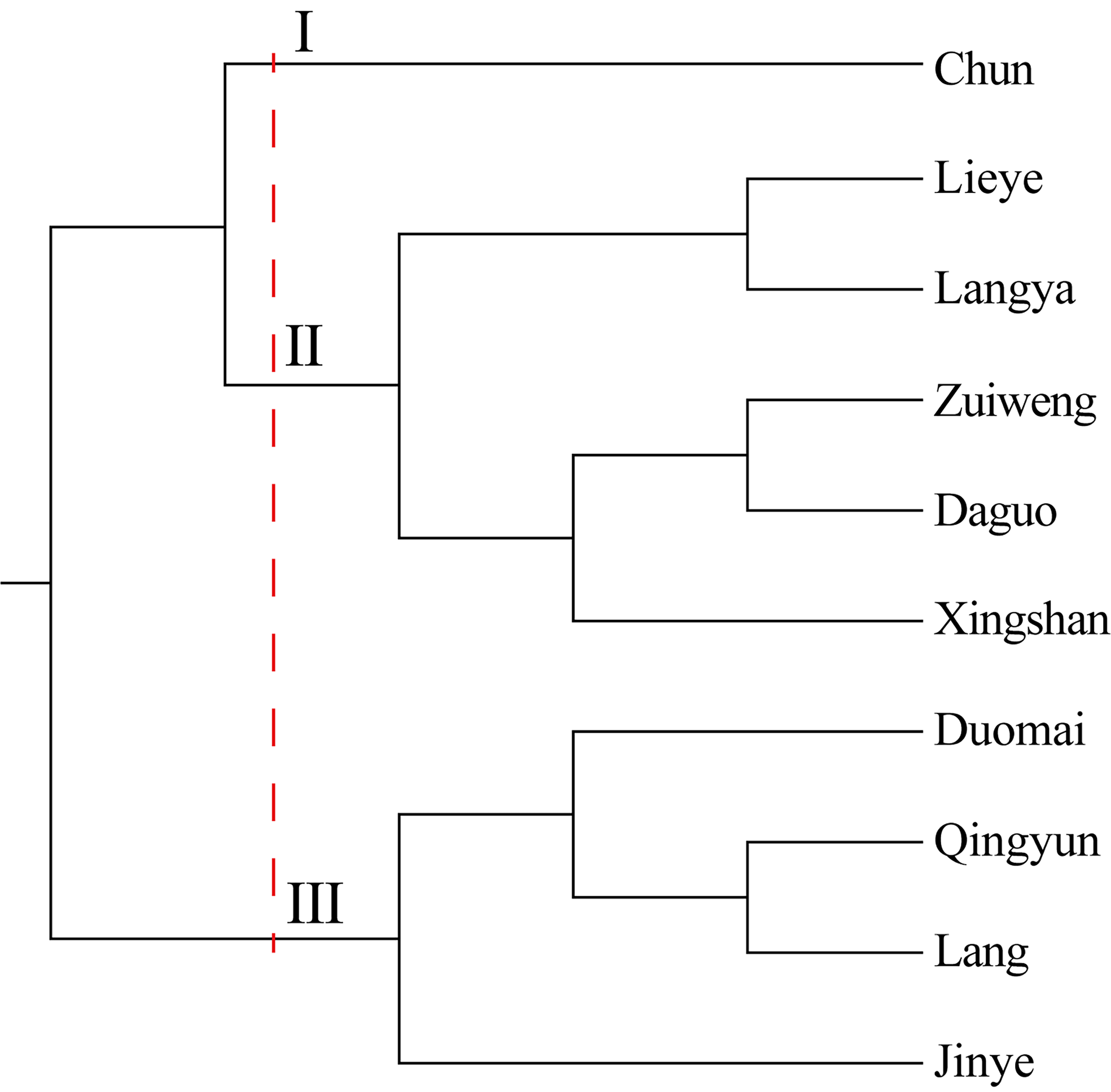


**Fig. S3** Evolutionary tree based on the leaf surface microstructures of ten *Ulmus* lines.


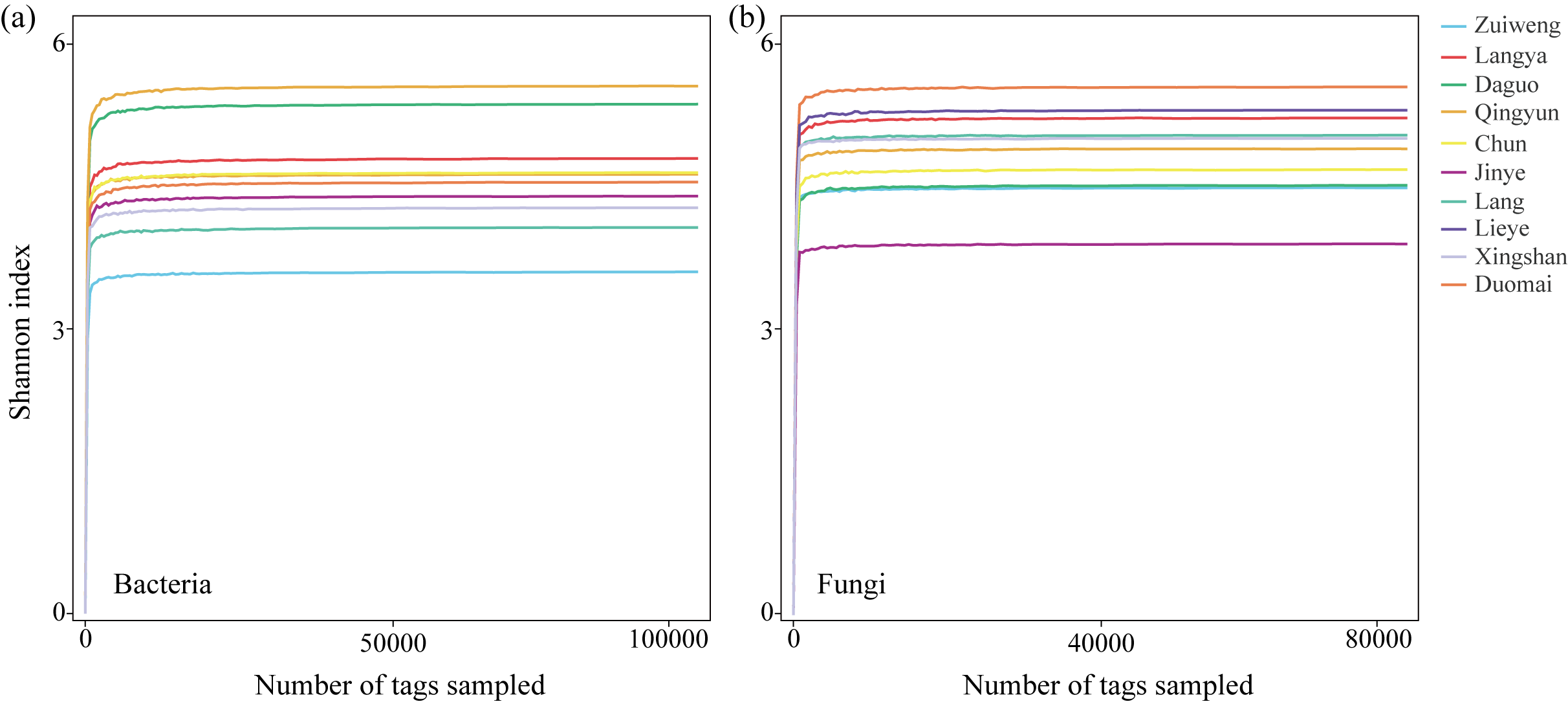


**Fig. S4** Rarefaction curves of bacterial (**a**) and fungal (**b**) communities.


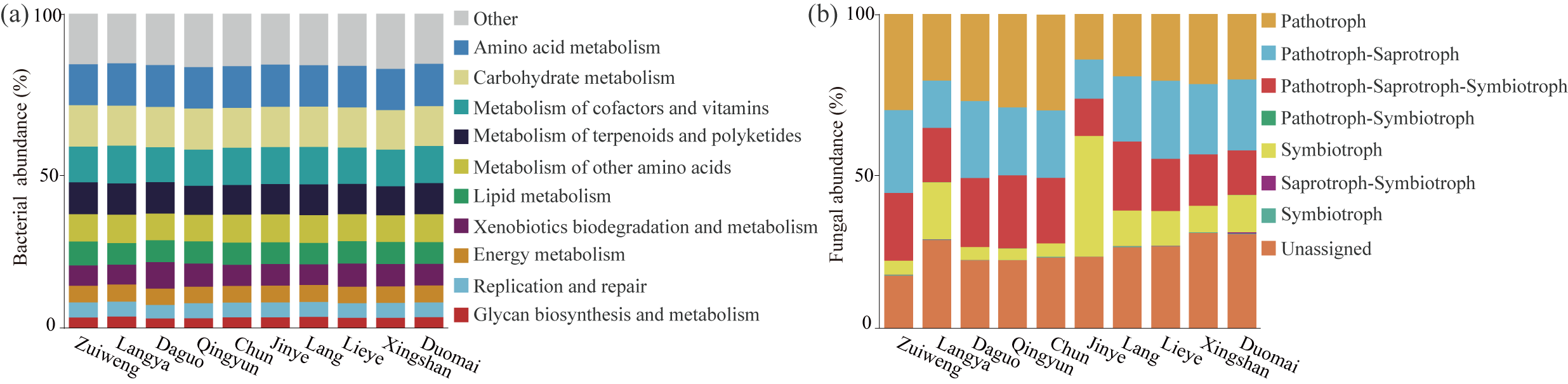


**Fig. S5** Phyllosphere microbial function predictive histograms at the bacterial (**a**) and fungal (**b**) scales.

**
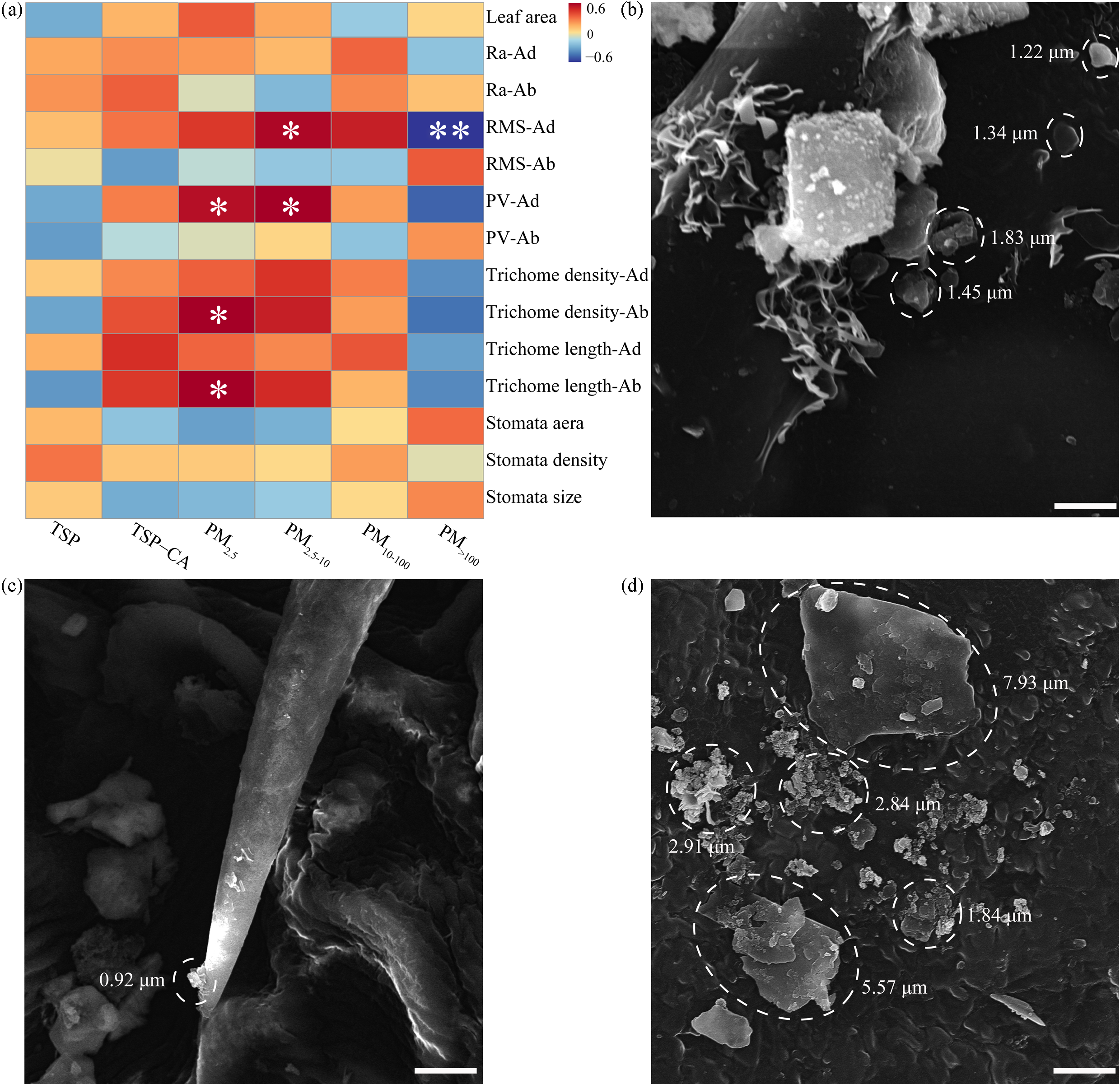
**

**Fig. S6** Correlation between and scanning electron microscopy (SEM) analysis of the microstructure features of the leaf surface and leaf-retained particulate matter (PM) indexes among ten *Ulmus* asexual lines. (**a**) correlation heat map; (**b**–**d**) SEM images of the different PM sizes retained on the leaf surface. *, and ** in (**a**) represent a significant correlation at *p* < 0.05 and *p* < 0.01, respectively. The scale bar is 2 μm in (**b**–**d**). Ab, abaxial; Ad, adaxial; PV, peak and valley value; Ra, profile arithmetic average error; RMS, raw roughness; TSP, total suspended particulate.


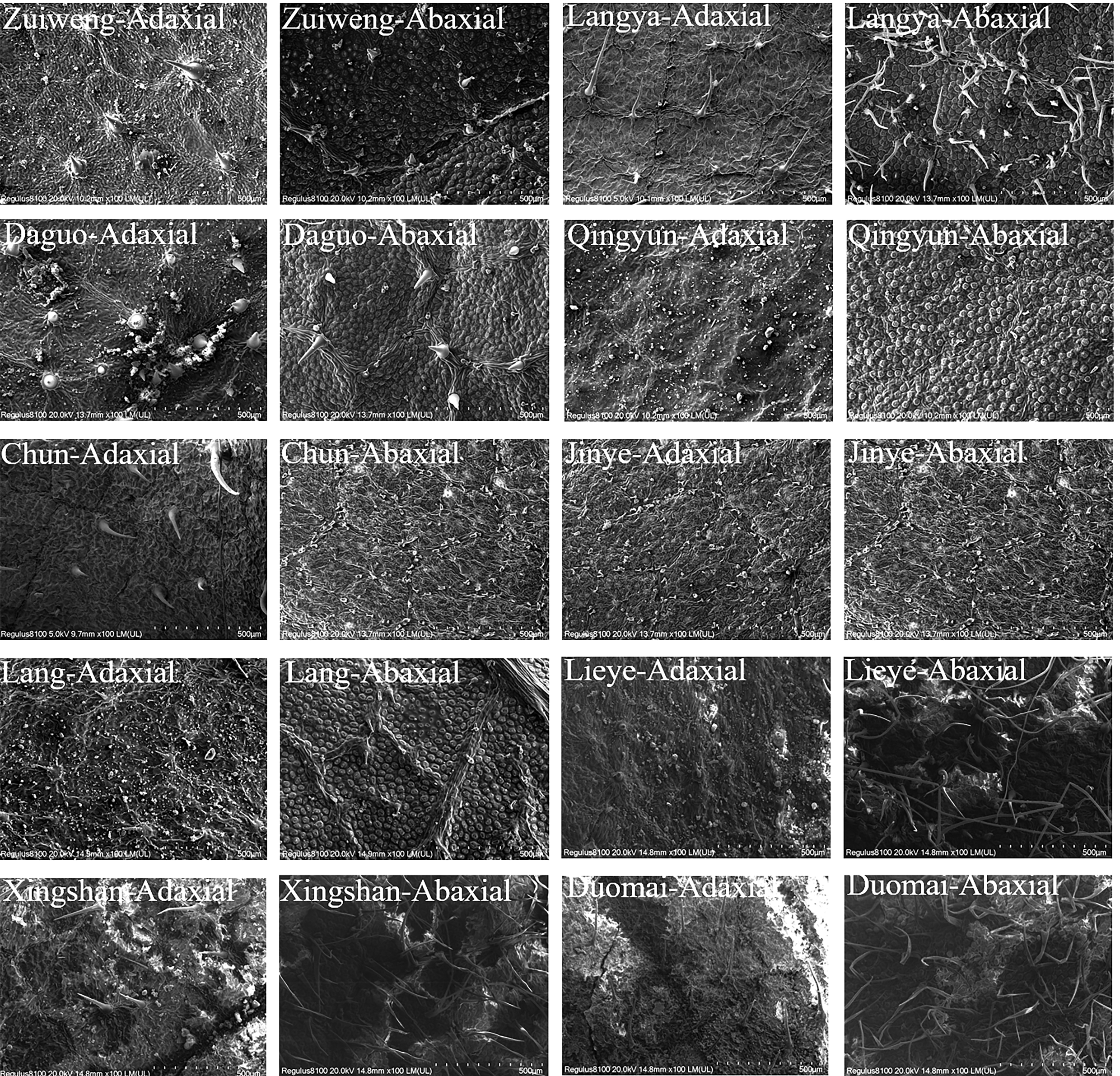


**Fig. S7** Scanning electron microscopy images (100×) of the unrinsed leaf surfaces of ten *Ulmus* asexual lines.
